# Tailored homo- and hetero- lanthanide porphyrin dimers: a synthetic strategy for integrating multiple spintronic functionalities into a single molecule†
†Electronic supplementary information (ESI) available: Synthesis and characterization of new compounds. CCDC 1565176–1565188. For ESI and crystallographic data in CIF or other electronic format see DOI: 10.1039/c8sc03762k


**DOI:** 10.1039/c8sc03762k

**Published:** 2018-10-19

**Authors:** Jennifer J. Le Roy, Jonathan Cremers, Isabel A. Thomlinson, Michael Slota, William K. Myers, Peter H. Horton, Simon J. Coles, Harry L. Anderson, Lapo Bogani

**Affiliations:** a Department of Materials , University of Oxford , 16 Parks Rd , OX1 3PH , Oxford , UK . Email: lapo.bogani@materials.ox.ac.uk; b Department of Chemistry , University of Oxford , Chemistry Research Laboratory , Mansfield Road , Oxford OX1 3TA , UK; c Centre for Advanced ESR , Department of Chemistry , University of Oxford , Inorganic Chemistry Laboratory , South Parks Road , Oxford , OX1 3QR , UK; d National Crystallography Service , School of Chemistry , University of Southampton , Southampton , SO17 1BJ , UK

## Abstract

We present the design, synthesis and magnetic properties of molecular magnetic systems that contain all elements necessary for spin-valve control in molecular spintronic devices in a single molecule. We investigate the static and dynamic magnetic properties and quantum spin properties of butadiyne-linked homo- and hetero-nuclear lanthanide-porphyrin dimers. A heterometallated porphyrin dimer containing both Tb^III^ and Dy^III^ centres is created rationally by the stepwise oxidative homocoupling of distinct lanthanide-porphyrin monomers. Tb^III^ and Dy^III^ mononuclear porphyrin complexes, homodimers and heterodimers all exhibit slow magnetic relaxation below 10 kelvin under a static magnetic field. The coherence times for Gd^III^ porphyrin monomers and dimers are found to be in excess of 3.0 μs at 2 K, allowing distinct magnetic manipulations in low temperature transport experiments.

## Abstract 2

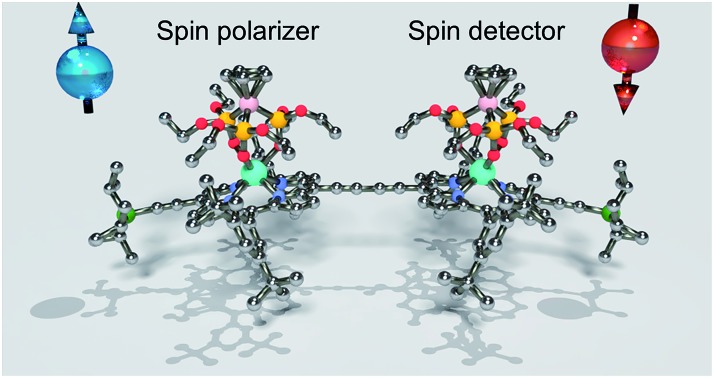
We present molecular magnetic systems that contain all elements necessary for spin-valve control in molecular spintronic devices in a single molecule.

## Introduction

Covalently linked porphyrin oligomers constitute a thriving area of research, particularly as model systems for light harvesting, molecular electronics and catalysis.^1^ Hetero-metallic porphyrin systems have been studied as donor–acceptor dyads.^2^ They would also be extremely appealing for the creation of molecular spintronic devices,^3^ which are typically composed of a spin polariser and a spin analyser, setting and reading out the current, respectively. The ultimate miniaturisation limit of these technology lies in the atomic and molecular level, and there is considerable research effort aimed at reducing spintronic elements down to the single-molecule level.^4^ The magnetic complexes integrated into molecular spintronic devices have mainly been single-molecule magnets (SMMs), with a particular role played by bis(phthalocyaninato) rare earth sandwich complexes.^5^ At sufficiently low temperatures, SMMs exhibit molecular hysteresis and behave as magnetic switches that can assume two classical states with a long relaxation time from one to the other.^6^ The observed slow relaxation could thus allow integrating the spintronic analyser and polariser inside a single molecule.

The development of a molecular spin valve requires the synthesis of rationally designed *ad hoc* systems that contain two different lanthanide-based SMMs. Recent advances in the synthesis of lanthanide hetero-dinuclear complexes have yielded prototypes for two-qubit molecular spin processors, which require coupled lanthanide centres.^7^ In contrast, for the aims of a molecular spin-valve, the spin centres must be magnetically shielded from one another, so as to operate independently. Moreover, each of the magnetic centres needs to be in contact with an electronic quantum dot, in order to optimise the spintronic transport signal. For these purposes the highly developed chemistry of porphyrin systems^8^ including homo-lanthanide dimers,8f–h may offer the degree of flexibility and reliability that is necessary for the rational tailoring of the magnetic molecules.

However, the use of metalloporphyrin oligomers in molecular magnetism^9^ is only scarcely investigated. Moreover, the creation of reliably hetero-dinuclear lanthanide complexes, requires the layout and implementation of a new methodology, in order to produce an original and interesting architecture.^10^ In particular, it is necessary to build a synthetic route that includes the introduction of protecting groups of very different polarity onto the mononuclear building blocks. Only in this way the subsequent separation by chromatography of the hetero-dinuclear assemblies becomes possible, in a reliable procedure.

Here we present the first synthetic approach to the rational creation of such molecular systems, concentrating on hetero-metallic rare-earth based porphyrin dimers. We analyse the magnetic properties of the independent building blocks and then concentrate on Dy–Tb dimers, where each lanthanide component acts as an individual magnetic source. The synthetic strategy is optimised to retain the presence of two electronic quantum dots in series, in the overall linear arrangement necessary to bridge electronic nanogaps. We investigate the quantum coherent properties of these molecular systems, paving the way to the creation of coherent spintronic devices. By pulsed electron paramagnetic resonance (EPR) we quantify the spin coherence times of Gd-based monomers and dimers, the magnetic interactions and the quantum features relevant for coherent manipulation.

## Results and discussion

### Molecular design and synthesis

Molecules for molecular spin valves need to possess two magnetic elements that can be tuned by an external magnetic field and can retain their magnetisation state for times as long as the measurement. Different conductivities can be obtained when the spins of the metal centres adopt a parallel or antiparallel configuration. This would constitute a fundamental, conceptual step forward in miniaturising devices to a truly molecular level. In addition, this strategy has the advantage of reducing the number of costly nanofabrication steps, by removing the need of magnetic electrodes.

The main requirements that new systems need to fulfil are rather stringent: the magnetic centres need to be reliably slow-relaxing, *i.e.* they must both have spin-flip times (*T*_1_) comparable to the experimental timescale (*i.e.*>10 s) in the temperature range used for single-molecule transport measurements (sub-K, for our purposes); high magnetic anisotropy barriers; the molecule must allow electrical conductance *via* single-electron processes, so that the molecular electronic quantum dots must be easy to reduce or oxidise; on the contrary, the spin centres must not easily undergo oxidation–reduction reactions, in order to preserve the magnetic state while in operation; the spin centres must be different, and show different coercive fields; the quantum dots must be connected to the spin centres; the system must be stable enough to permit deposition on different surfaces; it is desirable that a coherent counterpart can also be created, with dephasing times (*T*_2_) as long as possible.

**Fig. 1:**
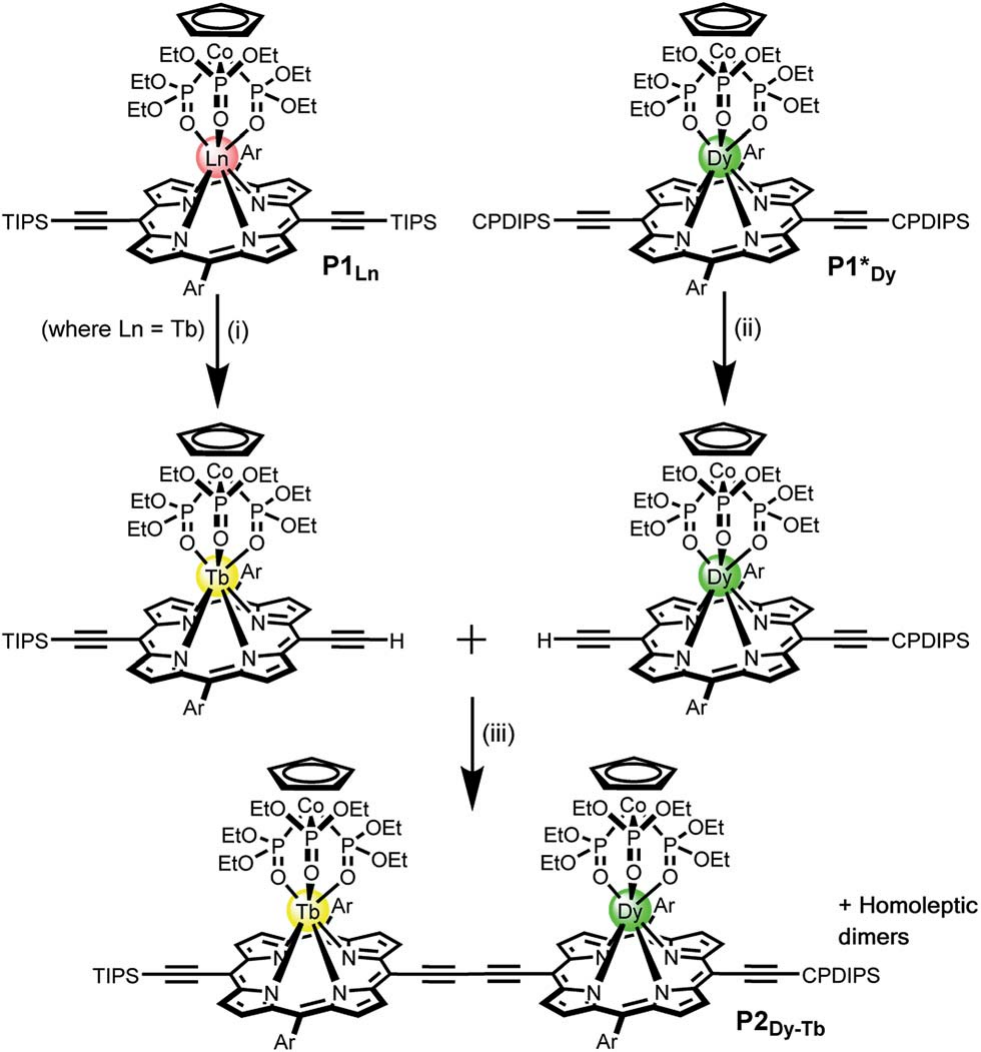
General structure of **P1_Ln_** and the synthetic route of the heterometallated dimer **P2_Dy-Tb_** (Ln = Tb). Reaction conditions: (i) TBAF, CHCl_3_/CH_2_Cl_2_, 50%; (ii) TBAF, CHCl_3_/CH_2_Cl_2_, 44%; (iii) Pd(PPh_3_)_2_Cl_2_, CuI, 1,4-benzoquinone, toluene/^i^Pr_2_NH, 48%. Ln = Y, Gd, Tb, Dy; TIPS = triisopropylsilane; CPDIPS = cyanopropyldiisopropylsilane; Ar = 3,5-bis(*tert*-butyl)phenyl.

We use porphyrin ligands in order to exploit their rich chemistry,^8^ for their high chemical stability and tendency to crystallise (Fig. 1). Lanthanide insertion can be achieved, as previously reported,^9^ by heating the free-base porphyrin in the presence of an excess Ln^III^Cl_3_ (Ln = Y, Gd, Tb, Dy) in a melt of diphenylether and imidazole. While many reports in the literature^8^ use molten imidazole as a solvent, diphenylether was an essential co-solvent for dissolving our porphyrins. Lanthanides are most commonly found in the +3 oxidation state and tend to adopt high coordination numbers both in the solid state and in solution due to their large ionic radii. Since porphyrins form di-anionic ligands which coordinate to metal ions through four nitrogen atoms, the Ln^III^ ions are not satisfied by a single porphyrin ligand, and one or more supplementary ligands are required in the design of the molecule to balance the charge and account for the remainder of the coordination sphere.

For this work we used Kläui's tripodal ligand [CpCo{P(=O) (OEt)_2_]_3_]^–^ (Cp = cyclopentadienyl) (Fig. 1) as a redox-stable capping group for the porphyrins.9c,d The tridentate ligand has three coordinating oxygen atoms and a single negative charge, yielding a neutral 7-coordinate lanthanide complex. This choice of ligands is fundamental to the creation of a reliable system that can operate in the non-crystalline state and in molecular electronic devices on surfaces. The tripodal shape of the Kläui ligand effectively shields the Ln^III^ from the environment. Moreover it confers solubility in most organic solvents, and its redox state and binding to Co(iii) are resilient to a broad range of reaction and purification conditions. All these characteristics are required if this complex were to be grafted into a devices. Moreover the ligand has an encumbered geometry that is likely to favour grafting onto surfaces *via* the porphyrin system, thus facilitating the presence of a single geometry in the devices.

**Fig. 2:**
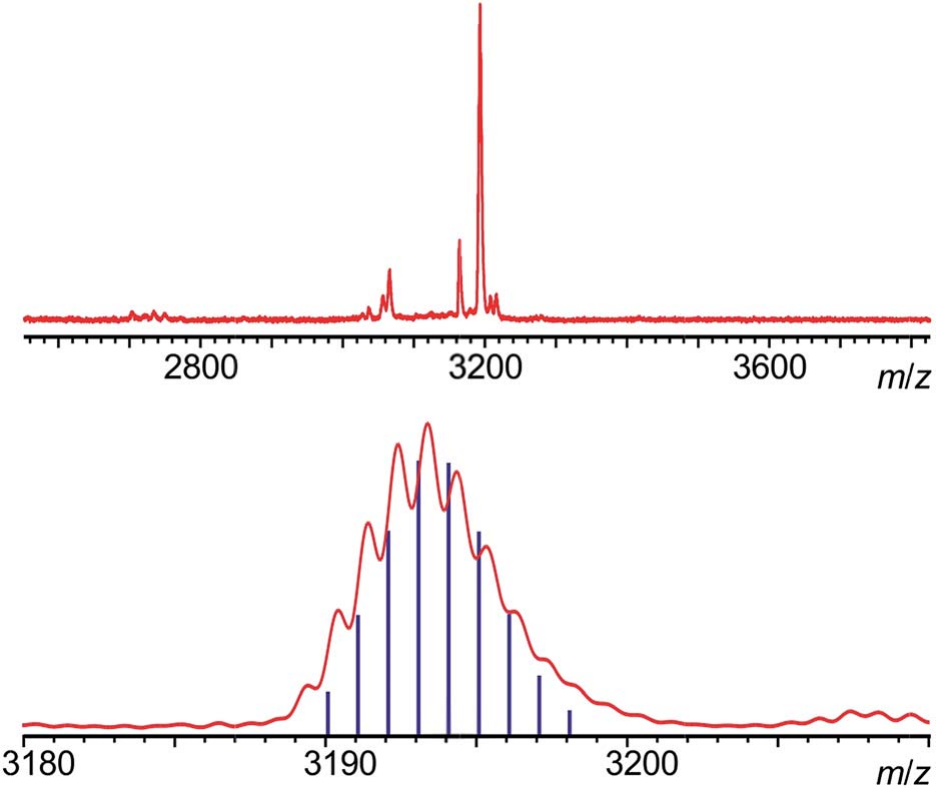
MALDI-TOF mass spectrum of **P2_Dy-Tb_**, acquired with a dithranol matrix. The absence of homodinuclear species (which would fall at 3188 and 3198 amu for homo-nuclear Tb^III^ and Dy^III^ complexes) is clearly observable (top), and the agreement of the isotopic pattern with the one expected for **P2_Dy-Tb_** is shown in the bottom.

Statistical de-protection of the porphyrin monomers (**P1_Ln_**), followed by oxidative homo-coupling, was used for the formation of the homoleptic dimers (**P2_Ln2_**).^11^ We prepared homoleptic dimers containing two Gd, Tb and Dy centres. The hetero-metallated dimer containing both terbium and dysprosium centres was prepared *via* an alternative route. An analogue of the dysprosium monomer (P1*_Dy_) was prepared containing polar CPDIPS protected acetylenes. Oxidative coupling of a mixture of mono de-protected **P1_Tb_** and P1*_Dy_, resulted in a statistical mixture of three dimers which could be separated on silica based on the different polarity of the protecting groups. Successful separation was confirmed using MALDI (Fig. 2) where the hetero-dinuclear peak is clearly present with the expected isotopic pattern, and homo-dinuclear species are not present. This indicates that no metal switching occurs during the oxidative coupling of monomer units.

The presence of the paramagnetic lanthanide ions renders the ^1^H-NMR spectra of the complexes rather uninformative. A diamagnetic yttrium analogue (**P1_Y_**) was prepared in order to confirm the general structure by NMR spectroscopy (ESI†).

### Structural characterisation

Single-crystals of the porphyrin monomers were grown by slow liquid diffusion of methanol into a CH_2_Cl_2_ solution of **P1_Ln_**. The homoleptic dimers (**P2_Ln2_**) yielded single crystals by slow evaporation of a solution of **P2_Ln2_** in a mixture of ethanol/CH_2_Cl_2_.

Single-crystals of each **P1_Y_**, **P1_Gd_**, **P1_Tb_**, **P1_Dy_**, **P2_Gd2_**, **P2_Dy2_** and **P2_Tb2_** were obtained and select crystallographic information is provided in Table 1. **P1_Gd_**, **P1_Tb_**, **P1_Dy_** and **P1_Y_** monomers display the same overall molecular connectivity but are not isostructural, with differing packing between the monomers resulting in the adoption of different space groups. **P1_Gd_** crystallizes in monoclinic *Cc*, **P1_Tb_** and **P1_Y_** crystallise in monoclinic *P*2_1_/c and **P1_Dy_** crystallises in a triclinic *P*1[combining macron] space group. Of these complexes, only the packing structure of **P1_Tb_** contains solvent molecules: one dichloromethane molecule per Tb complex. It is surprising that these monomer complexes crystallise differently because they were all crystallised under identical conditions. Additionally, for **P1_Dy_** there are two molecules in the asymmetric unit whereas all other monomers have one molecule per asymmetric unit. In related lanthanide structures9*e* the Gd^III^, Tb^III^ and Dy^III^ complexes all crystallise in a monoclinic *P*2_1_/c space group. The discrepancy in packing arrangement is a manifestation of crystal polymorphism.^12^

**Table 1:** Selected crystallographic angles (°) and distances (Å)

	Space group	Nearest intramolecular Ln^III^–Ln^III^ (Å)	Nearest intermolecular Ln^III^–Ln^III^ (Å)	Porphyrin_Centroid–_Ln^III^–Co^III^ (°)	Porphyrin_Centroid–_Ln^III^ (Å)	Ln^III^–P or 1–P or 2–Ln^III^ torsion (°)	Co^III^–Ln^III^ (Å)
**P1_Y_**	*P*2_1_/c	—	11.1407(3)	177.83(4)	1.1958(9)	—	4.2699(4)
**P1_Gd_**	*Cc*	—	12.10900(9)	176.97(5)	1.2360(11)	—	4.3042(4)
**P1_Tb_**	*P*2_1_/*c*	—	9.87989(19)	174.50(13)	1.233(3)	—	4.2467(14)
**P1_Dy(A)_**	*P*1[combining macron]	—	9.8418(3)	170.48(9)	1.2084(19)	—	4.2457(8)
**P1_Dy(B)_**	*P*1[combining macron]		9.8418(3)	175.49(9)	1.212(2)		4.2410(8)
**P2_Gd2_**	*P*1[combining macron]	13.9202(7)	10.3106(2)	174.15(10)	1.266(2)	180.0	4.272(3)
**P2_Dy2_**	*P*1[combining macron]	13.8726(6)	10.25340(18)	174.63(7)	1.2239(16)	180.0	4.248(2)
**P2_Tb2_**	*P*1[combining macron]	13.9299(5)	10.3082(1)	174.47(9)	1.2519(18)	180.0	4.270(3)
GdP L_OEt_^–^^*a*^ 8*e*	*C*2/*c*	—	10.14	177	1.23	—	4.30
TbP L_OEt_^–^^*a*^ 8*e*	*C*2/*c*	—	10.11	177	1.23	—	4.29
DyP L_OEt_^–^^*a*^ 8*e*	*C*2/*c*	—	10.10	177	1.22	—	4.28
HoP L_OEt_^–^^*a*^ 8*e*	*C*2/*c*	—	10.14	177	1.23	—	4.30

^*a*^Data from ref. 9*e*: LnP L_OEt_^–^ = [(L_OEt_)Ln-(TPP)]·0.25H_2_O where TPP = 5,10,15,20-tetraphenylporphyrinate, L_OEt_^–^ = [(*η*^5^-C_5_H_5_)Co{P(=O) (OEt)_2_}_3_]^–^.

**Fig. 3:**
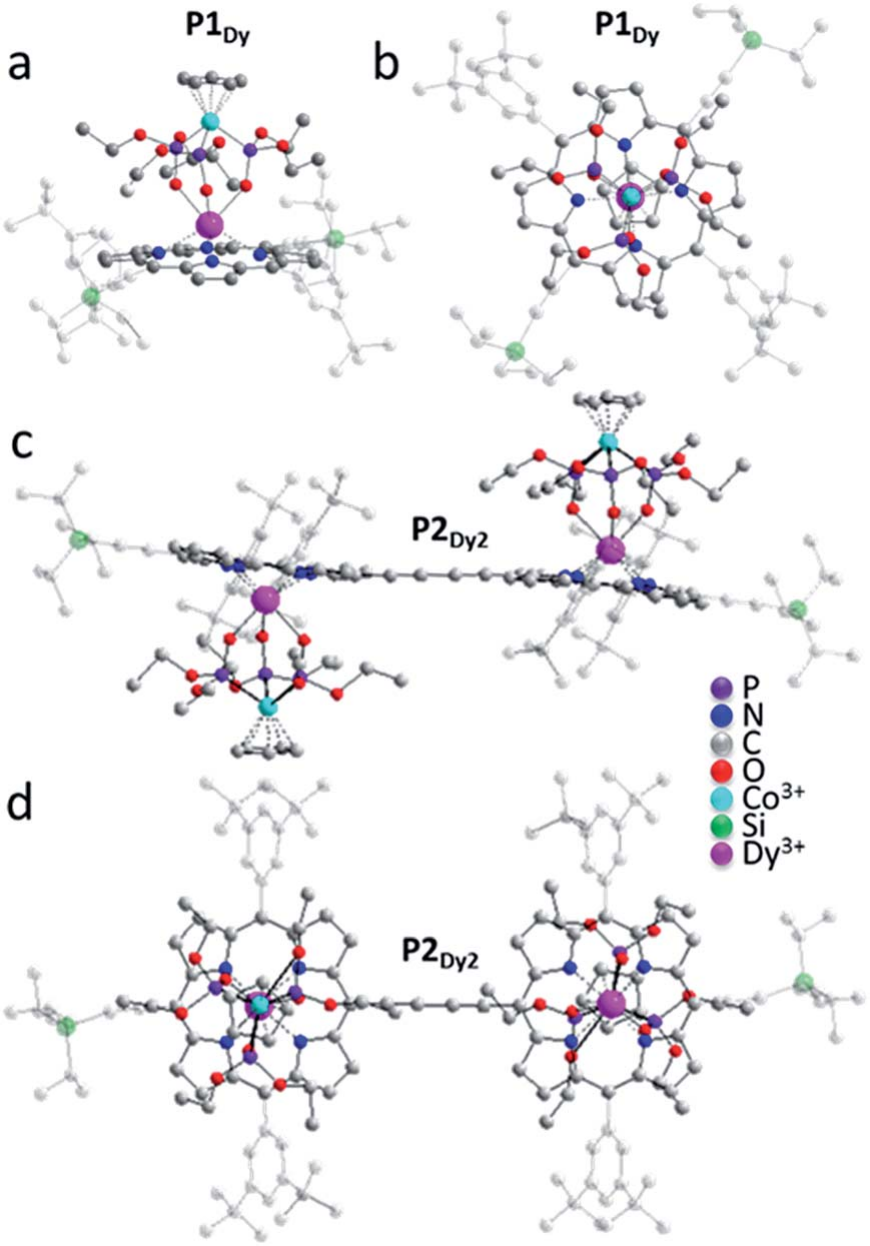
Crystal structures of representative examples of the investigated compounds, as acquired at room temperature. (a) Side view of **P1_Dy_**; (b) top view of **P1_Dy_**, along the Co–Dy vector; (c) side view of **P2_Dy2_**; (d) top view of **P2_Dy2_**. H atoms have been omitted for clarity. Triisopropylsilylethynyl and aryl groups are faded for clarity and the colour-scale is: carbon = light grey, oxygen = red, nitrogen = dark blue, phosphorous = purple, cobalt = light blue and dysprosium = pink.

For simplicity only one monomer of the Dy-analogue will be described in detail, complete crystallographic data for all the compounds are available in the ESI.† For **P1_Dy_** the Dy^III^ ion is sandwiched between four N atoms of the porphyrin ligand and three O atoms of the capping ligand resulting in a 7-coordinate complex (Fig. 3a). The two aryl pendants on the porphyrin are not parallel to the plane of the porphyrin, but are tilted at an angle of 67.42(16)° and 87.74(14)°. The Dy^III^ ion sits 1.212(2) Å out of the plane of the porphyrin and 4.2410(8) Å from the Co^III^ ion. The Cp ring and porphyrin appendage are near parallel to each other with a tilt angle of 4.4(3)°. The porphyrin, Dy^III^ and Co^III^ ions also have a near-linear arrangement with a porphyrin-centroid (C_Por_)–Dy–Co angle of 175.49(9)°, illustrated in Fig. 3a and b.

Examination of the four lanthanide monomers reveals that the C_Por_–Ln distance for **P1_Gd_**, **P1_Tb_**, **P1_Dy_** and **P1_Y_** decreased roughly in proportion to the atomic radius from **P1_Gd_** through **P1_Y_**. The different packing arrangement may explain why we do not observe any periodic trends related to intermolecular Ln–Ln (Å) distance or linearity of the complexes measured through the C_Por_–Ln–Co tilt angle (Table 1).

We next examined the solid state structures of three homoleptic lanthanide dimers. In contrast to the monomers, each of **P2_Gd2_**, **P2_Tb2_** and **P2_Dy2_** complexes crystallise in a triclinic *P*1[combining macron] space group with no solvent molecules in the crystal lattice and all structures contain a centre of inversion. The nearest intramolecular Ln–Ln distance decreases moving from left to right across the lanthanide series **P1_Gd_** –13.9202(7) Å, **P1_Tb_** –13.9299(5) Å and **P1_Dy_** –13.8726(6) Å. There is a centre of inversion at the centre of each porphyrin dimer. This is reflected in the Ln–C_Por_–C_Por_–Ln torsion angle (180° in each dimer). We expect that the centre of inversion would not be maintained in solution or on a surface as the barrier to torsional rotation in a butadiyne-linked porphyrin dimer is quite low (Δ*H* = 5.27 kJ mol^–1^).^13^ There is very little deviation of the coordination environment surrounding the lanthanide ions in the monomer and dimer structures. SHAPE^14^ software was used to compare the polyhedral of **P1_Dy_**, **P2_Dy2_**, **P1_Tb_**, **P2_Tb2_**, **P1_Gd_**, **P2_Gd2_** with other possible 7-vertex polyhedral. All 6 complexes exhibited predominately either capped octahedron or a capped trigonal prism (see full discussion in the ESI†).

The main objective of creating the **P2_Dy-Tb_** is to obtain hetero-dinuclear systems where every molecule in the crystal contains one Dy^III^ centre and one Tb^III^ centre. We demonstrated the purity of this hetero-dimer using Matrix Assisted Laser Desorption Ionization-Time of Flight (MALDI-TOF) mass spectrometry, which is an excellent way of identifying perfectly hetero-dinuclear compounds, as they provide a starkly different mass value and isotopic pattern for hetero and homo-dinuclear molecules (Fig. 2).

### Magnetic properties

Each of **P1_Gd_**, **P1_Tb_**, **P1_Dy_**, **P2_Gd2_**, **P2_Dy2_**, **P2_Tb2_** and **P2_Dy-Tb_** have an overall charge of zero. The Co^III^ ion is diamagnetic and the magnetic properties discussed below originate solely from the localized spin of the Ln^III^ ion(s). We shall investigate both the static and dynamic properties of the systems, relevant to their suitability as spin-valve components.

#### Static magnetic properties

**Fig. 4:**
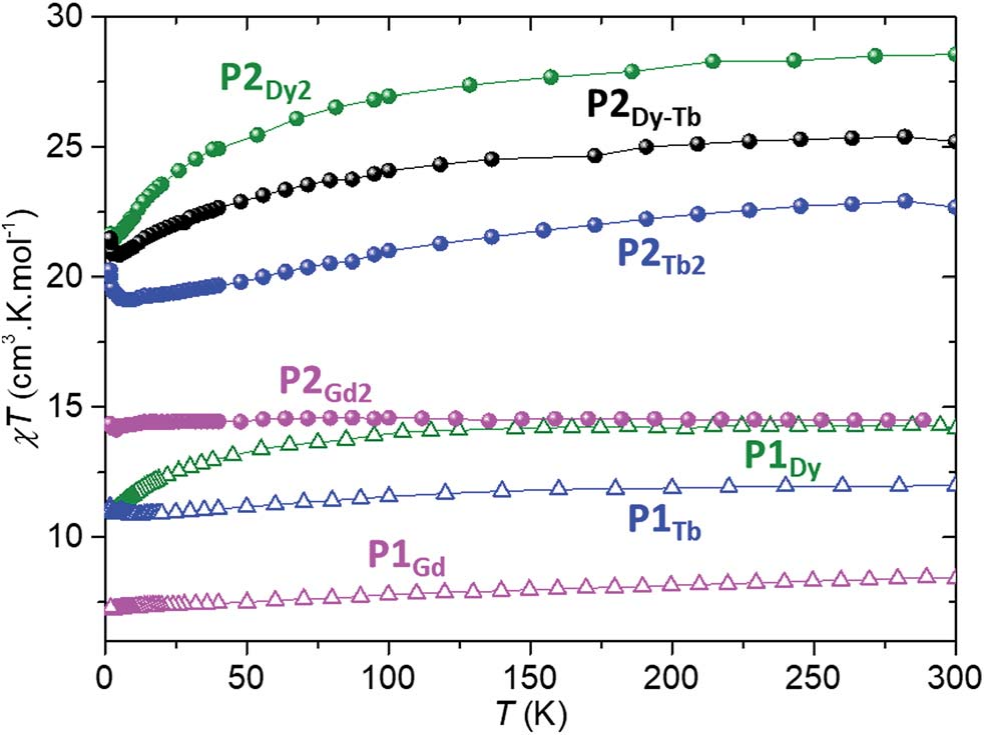
Temperature dependence of the static magnetic susceptibility times the temperature, for all lanthanide-porphyrin complexes described in the text. Spheres indicate dinuclear compounds, open triangles mononuclear ones; Gd compounds are in pink, Dy in green, Tb in blue and the hetero-dinuclear one in black. All measurements were performed on crystalline powder samples under an external static magnetic field of 1 kOe.

The variable temperature magnetic properties of all complexes were determined using a MPMS-XL SQUID magnetometer. Fig. 4 provides the static magnetic properties for each crystalline complex, with the magnetic susceptibility *χ* calculated as the ratio between the magnetisation *M* and the applied magnetic field *H*. The *χT* products for the **P1_Gd_**, **P1_Tb_** and **P1_Dy_** monomers are 8.40, 11.19 and 14.16 cm^3^ kmol^–1^ respectively at room temperature. These values are in good agreement with the theoretical values of 7.88, 11.82 and 14.17 cm^3^ kmol^–1^ for a single Gd^3+^ ion (^8^S_7/2_, S = 7/2, L = 0, g = 2), a Tb^3+^ ion (^7^F_6_, S = 3, L = 3, g = 3/2) and a Dy^3+^ ion (^6^H_15/2_, S = 5/2, L = 5, g = 4/3), respectively. The overall shape of each variable temperature curve provides further information about the magnetic properties of each compound. The ^8^S_7/2_ ground state of Gd^III^ has zero orbital angular momentum, and the straight line is consistent with its isotropic nature. Conversely, the deviation from linearity observed for dysprosium at low temperatures is to be expected as a result of the large magnetic anisotropy of Dy^III^, with its 4f^9^ configuration and a ^6^H_15/2_ ground state. Interestingly, at low temperature Tb^III^ demonstrates a slight increase in the *χT* product below 10 K. This probably indicates an intermolecular ferromagnetic interaction between neighbouring spin carriers (closest Tb–Tb intermolecular distance 9.9 Å).

The room temperature *χT* values for each **P2_Gd2_**, **P2_Tb2_**, **P2_Dy2_** and **P2_Dy-Tb_** dimers are 14.50, 22.69, 28.56 and 25.22 cm^3^ kmol^–1^ and are in good agreement with twice the theoretical values of monomers 15.76, 23.64, 28.34 and 25.99 cm^3^ kmol^–1^ respectively. For **P2_Gd2_** the *χT* product remains basically unchanged from room temperature to 5 K, with only a very slight decrease below 5 K. This agrees well with an S = 7/2 centre with no anisotropy and with very small anti-ferromagnetic interactions. For **P2_Tb2_** and **P2_Dy2_** the *χT* product decreases steadily from 300 K down to 5 K, only to eventually increase again below 5 K. In this case, the marked decrease below 50 K is attributed primarily to the large inherent magnetic anisotropy of Dy^III^ and Tb^III^ ions, while the slight low temperature increase may indicate weak ferromagnetic coupling between intramolecular Ln^III^ ions. Such magnetic interactions can be dipolar or exchange-coupling in nature. The distances between Ln ions are presented in Table 1.

#### Incoherent dynamic magnetic properties

AC susceptibility studies of all Dy^III^ and Tb^III^ complexes revealed no out-of-phase zero field magnetic susceptibility in the *χ*′′ plot at 1.8 K, presumably due to the presence of ground state quantum tunnelling of the magnetisation (QTM). Under a dc field of 2000 Oe, full frequency and temperature dependent peaks are observed in the *χ*′′ susceptibility for all Dy^III^ and Tb^III^ complexes.

**Fig. 5:**
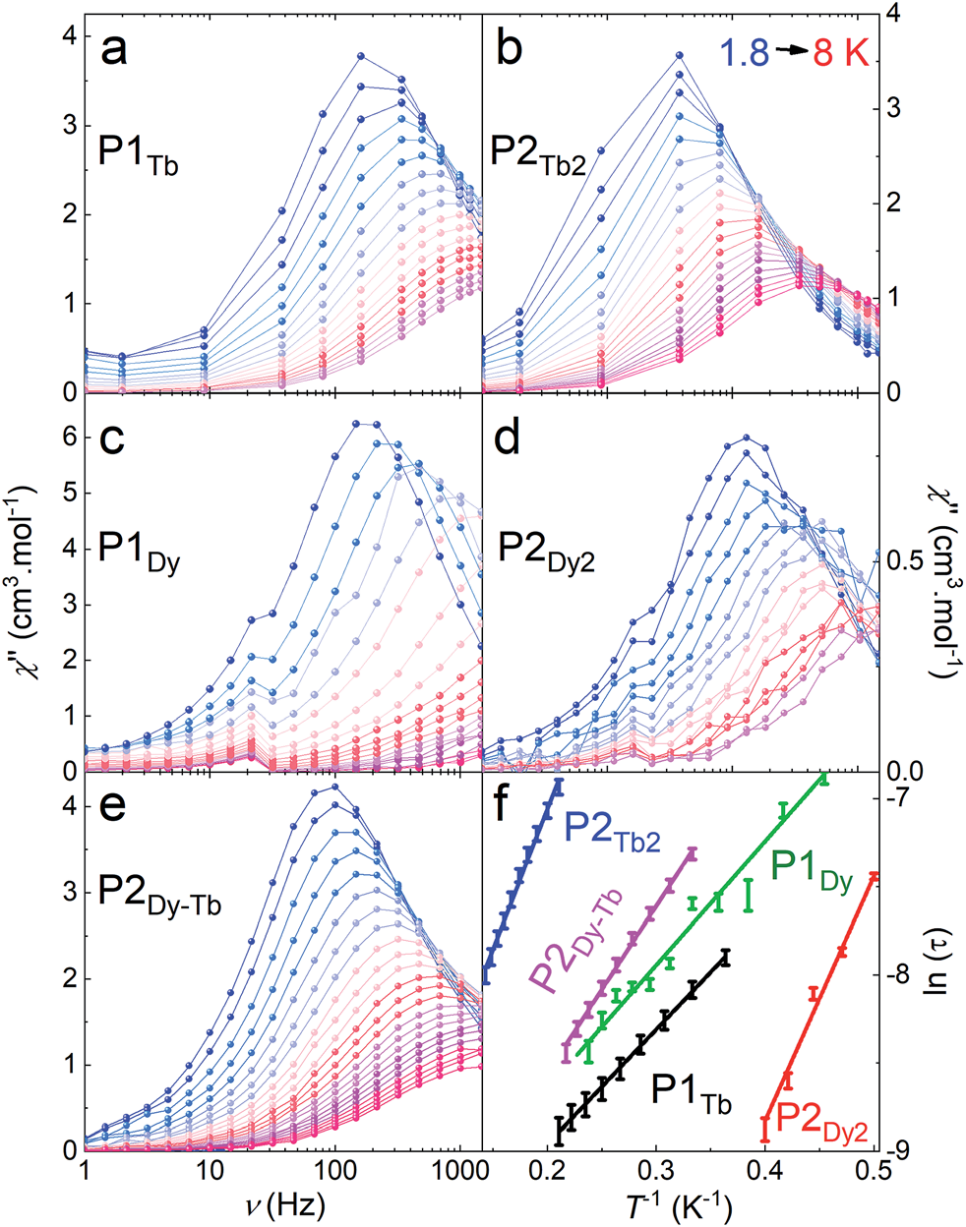
Dynamic magnetic susceptibility of crystalline samples of **P1_Tb_** (a), **P2_Tb2_** (b), **P1_Dy_** (c), **P2_Dy2_** (d) and **P2_Dy-Tb_** (e) for different temperatures (colour scale). All measurements were performed under a 2000 Oe constant magnetic field, in a 1–1500 Hz frequency range. (f) A plot of ln(*τ*) *versus* 1/*T* for all compounds, showing the Arrhenius behaviour. Data points are represented with error bars obtained from the fitting of the peak maxima of the frequency dependence in a–e with Lorentzian curves. Solid lines represent linear regression to the data.

The two monomeric systems, **P1_Tb_** and **P1_Dy_**, show very similar slow magnetic relaxation dynamics, with frequency dependent peaks that shift to higher frequencies on increasing *T* (Fig. 5a and c). Using the *χ*′′ peak maxima determined using a Lorentz fit, and the Arrhenius law (*τ* = *τ*_0_ exp(*U*_eff_/*k*_B_*T*) where *τ* = 1/(2π*υ*) and *υ* is the frequency corresponding to the maxima of each *χ*′′ peak), the effective energy barriers were obtained; **P1_Tb_**, *U*_eff_ = 7(2) K with a *τ*_0_ = 3.4 × 10^–5^ s and **P1_Dy_**, *U*_eff_ = 14(4) K with a *τ*_0_ = 2.5 × 10^–6^ s. The relaxation barrier and *τ*_0_ for **P1_Dy_** is very close to that reported for the similar 10,15,20-tetraphenylporphyrin dysprosium complex.9*e*

Homoleptic dimers **P2_Tb2_** and **P2_Dy2_** displayed slightly different magnetic properties to their corresponding monomer complexes: **P2_Tb2_**, *U*_eff_ = 15(2) K with *τ*_0_ = 3.6 × 10^–5^ s; **P2_Dy2_**, *U*_eff_ = 7(2) K with a *τ*_0_ = 4.5 × 10^–5^ s. The slight differences in the *U*_eff_ between mono- and bimetallic complexes is most likely a result of the slight change in the second coordination sphere where the TIPS pendent is replaced by an acetylene resulting in a less symmetric second coordination sphere. Changes to the second-coordination sphere can often influence the magnetic properties of lanthanide ions.^15^**P2_Dy-Tb_** also showed similar behaviour to the above with *U*_eff_ = 9(2) K with *τ*_0_ = 2.9 × 10^–5^ s confirming slow-magnetic relaxation at low temperatures.

#### Coherent dynamic magnetic properties

**Fig. 6:**
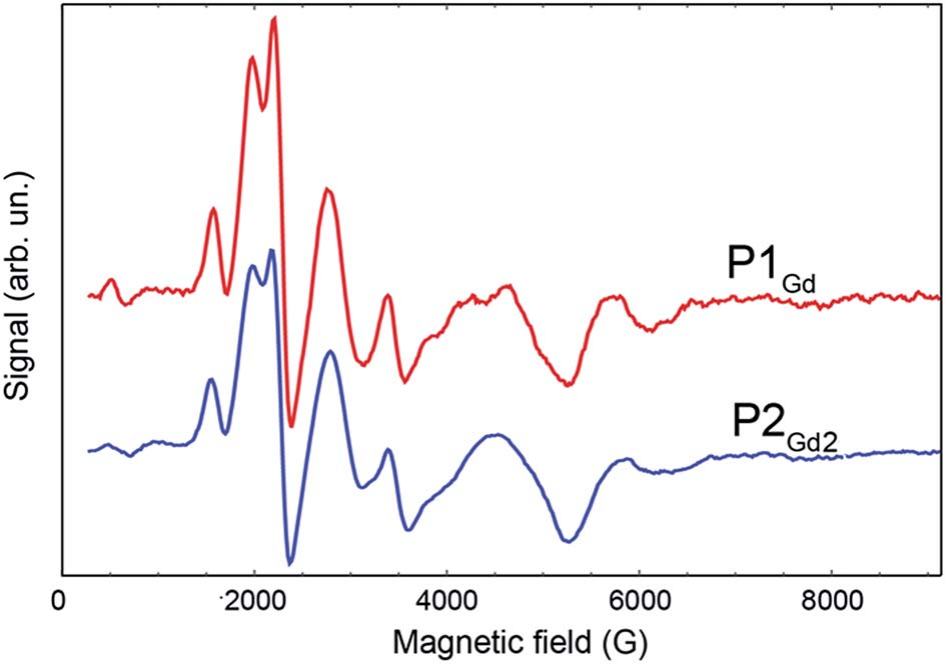
EPR spectra comparing the derivative of the free-induction-decay-detected absorption of **P1_Gd_** (red line) and **P2_Gd2_** (blue line). The spectra were acquired at X-band frequency (9.4 GHz) at 3 K in a 1 : 1 mixture of dichloromethane and toluene.

We accessed the quantum coherence properties of the systems by pulsed electron paramagnetic resonance (EPR) techniques. These can provide extremely valuable information on whether the compounds can be used for quantum operation processing *via* single-electron transport at low temperatures, which for example includes *T*_1_ and *T*_2_ times. By applying different sequences of microwave pulses, we can extract the spin-lattice relaxation time, *T*_1_, and the spin–spin dephasing (or phase-memory) time, *T*_M_. Furthermore, pulsed EPR techniques can provide valuable information on very low spin–spin dipolar and exchange interactions in dimer systems, as is valuable for the molecular spin-valves. We restrict our analysis to the compounds based on Gd^III^, **P1_Gd_** and **P2_Gd2_**, because of the extreme broadening and zero-field splitting of Dy^III^ and Tb^III^ centres due to spin–orbit interactions with their environment. As the orbital momentum is zero for the ground state, Gd^III^ systems can be treated as pure spin systems with a total spin of S = 7/2. Interactions and mixing with higher excited multiplets and higher-order spin–orbit interactions will be neglected here. The low symmetry crystal-fields of **P1_Gd_** and **P2_Gd2_** result in a complicated crystal-field splitting with four Kramers doublet eigenstates, where states with different magnetic quantum numbers *m*_s_ = ±1/2, ±3/2, ±5/2, ±7/2 are mixed. Fig. 6 shows the spectra obtained by the derivative of the free-induction-decay (FID) detected absorption at X-band. The broad linewidths, hundreds of Gauss, are typical of Gd^III^ EPR spectra.6*h* Comparison of **P1_Gd_** and **P2_Gd2_** reveals a very similar spectrum, with the additional Gd^III^ ion in the **P2_Gd2_** dimer producing only minimal changes around 2000 and 3300 Oe. The small changes in the spectrum can be due to weak dipolar and exchange interactions between the Gd^III^ ions, where the latter one is mediated by the alkyne bridge. Due to the broad linewidth, the interactions cannot be resolved by standard CW spectroscopy at X-band frequency. For improved resolution and to determine coherence times, we used pulsed EPR techniques.

**Fig. 7:**
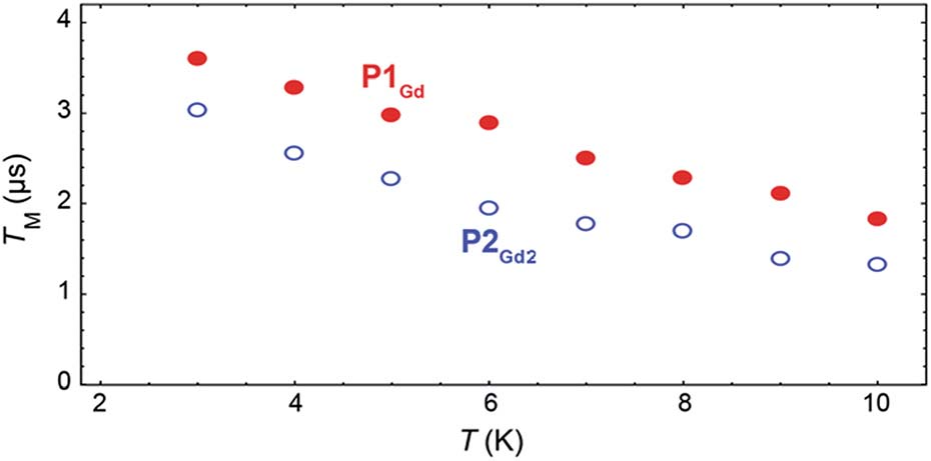
Temperature dependence of the quantum coherence time *T*_M_ for **P1_Gd_** (red full dots) and **P2_Gd2_** (blue empty dots), as measured at 344 mT at 9.37 GHz in a 1 : 1 mixture of dichloromethane and toluene.

The phase-memory times, *T*_M_, were determined using a variation of the Hahn-echo sequence (Fig. 7, ESI for details†). *T*_M_ is found to increase steadily on lowering the temperature, with an almost linear trend from 10 to 2 K, reaching 3.6 μs and 3.0 μs for **P1_Gd_** and **P2_Gd2_**, respectively. This indicates only a marginal decoherence time introduced by the dimerisation, and provides times that amply meet the requirements for possible quantum operations. The echo signal decays mono-exponentially *versus* the free evolution time *τ* of the Hahn echo (ESI†), indicating no spectral diffusion. Analogously, no detectable dependence of the *T*_2_ times on the magnetic field is observed (ESI†). This means that the dinuclear system will indeed appear composed of two distinct magnetic centres in low temperature transport experiments. Measurements of the single molecule transport and magnetic properties are currently underway.

## Conclusions

In conclusion, we have created hetero-dinuclear single molecule magnets based on porphyrin ligands, using a rational stepwise approach. We demonstrated the use of protecting groups of very different polarity for the selective creation of hetero-metallic porphyrin complexes. The protecting group methodology can now be used as a tool for the tuning of functional poly-porphyrin molecules, providing a route to hetero-dinuclear molecular magnets.^7^,^16^ From this perspective, the dimers here created are the first that are sufficiently stable to undergo purification steps such as chromatographic separations.

An extension of these novel synthetic approach would be to rationally assemble poly-heteronuclear lanthanide chains. For example, tetra- and hexa-heteronuclear chains with a pre-defined order of the magnetic centres are planned *via* stepwise repetition of the oxidative homo-coupling step. Including the optical properties of the porphyrin ligands themselves, future work will include addition of functional groups such as luminescent appendages, light antennae or anchoring groups.

The magnetic properties of the compounds created show the desired slow magnetic relaxation and the coherent properties indicate μs coherence times at low temperatures for Gd^III^ compounds. These compounds offer a testbed to probe magnetic interactions in molecular systems, and indicate a possible applicative direction for ring-shaped poly-porphyrins^17^ in spintronics and in single-molecule EPR investigations.^18^ All stringent requirements for the creation of spin-valves are simultaneously met and the new molecules show slow relaxation, magnetic anisotropy, the presence of molecular electronic quantum dots, different spin centres and stability that permits deposition on different surfaces.

## Conflicts of interest

There are no conflicts to declare.

## Supplementary Material

Supplementary information

Crystal structure data
