# Supplementary material for: Tailored homo- and hetero- lanthanide porphyrin dimers: a synthetic strategy for integrating multiple spintronic functionalities into a single molecule
Source: Chem Sci. 2018 Oct 19;9(45):8474–81. doi: 10.1039/c8sc03762k (PMC6256854; doi:10.1039/c8sc03762k)
Supplement: Supplementary file 1 [file SC-009-C8SC03762K-s001.pdf]

Electronic Supplementary Material (ESI) for Chemical Science.
This journal is © The Royal Society of Chemistry 2018

Electronic Supplementary Information

Tailored Homo- and Hetero- Lanthanide Porphyrin Dimers: a Synthetic Strategy for Integrating Multiple Spintronic Functionalities into a Single Molecule

Jennifer J. Le Roy,‡ [a] Jonathan Cremers,‡ [b] Isabel A. Thomlinson, [b] Michael Slota, [a] William K. Myers,[c] Peter H. Horton,[d] Simon J. Coles,[d] Harry L. Anderson[b] and Lapo Bogani[a]

[a]Department of Materials, University of Oxford, Parks Rd, Oxford, OX1 3PH, UK.
[b]Department of Chemistry, University of Oxford, Chemistry Research Laboratory, Mansfield Road, Oxford, OX1 3TA, UK.
[c]Centre for Advanced ESR, Department of Chemistry, University of Oxford, Inorganic Chemistry Laboratory, South Parks Road, Oxford, OX1 3QR, UK.
[d]National Crystallography Service, School of Chemistry, University of Southampton, Southampton, SO17 1BJ, UK.

Synthetic Details S2

X-ray Crystallography S23

SQUID Magnetometry S28

Electron Paramagnetic Resonance S30

References S34

1 Synthetic Details

1.1 General Experimental

Dry toluene and THF were obtained by passing the solvents through columns of alumina, under nitrogen. Diisopropylamine (*i*-Pr_2NH) was distilled from CaH_2 and kept over activated molecular sieves (3 Å, 8–12 mesh). Unless specified otherwise, all other solvents were used as commercially supplied. Flash chromatography was carried out on silica gel 60 under positive pressure. Analytical thin-layer chromatography was carried out on aluminum-backed silica gel 60 F254 plates. Visualization was achieved using UV light when necessary.

All UV-vis-NIR spectra were recorded in solution using a Perkin-Lambda 20 spectrometer (1 cm path length quartz cell).

Unless stated otherwise, $^1H/^{13}C$ NMR spectra were recorded at 298 K using a Bruker AV400 (400/100 MHz) instrument. ^1H, and ^{13}C NMR spectra are reported in ppm; coupling constants are given in Hertz, to the nearest 0.1 Hz. The solvent used was $CDCl_3$.

MALDI-ToF spectra were measured at the EPSRC National Mass Spectrometry service (Swansea) using the Applied Biosystems Voyager DE-STR or at the University of Oxford using Waters MALDI Micro MX spectrometer utilizing dithranol as a matrix.

1.2 Synthetic Procedures

The synthesis and characterization of zinc porphyrin **P1$_{Zn}$** and that of the capping ligand **Na[CpCo(P(O)(OEt)$_2$)$_3$]** (**NaL$_{OEt}$**) and its precursor **Cp$_2$Co$_3$(P(O)(OEt)$_2$)$_6$** have been reported previously.[1-6] The general reaction scheme to the heterometallated dimer **P2$_{Dy\text{-}Tb}$** is shown in Figure S1.

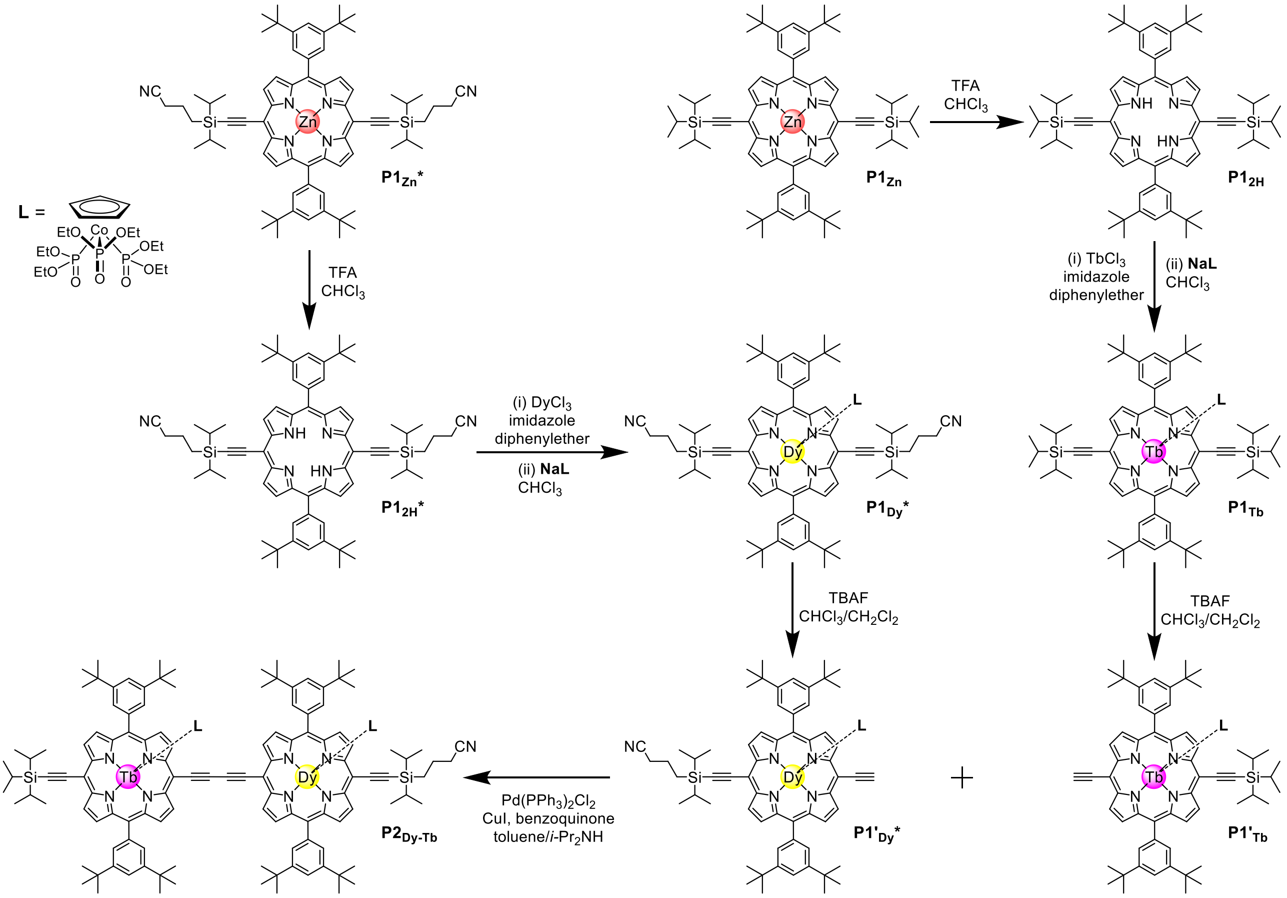

Figure S1: The general reaction scheme to **P2$_{Dy\text{-}Tb}$**.

P1$_{2H}$

P1$_{Zn}$ (2.12 g, 1.77 mmol) was dissolved in $CHCl_3$ (350 mL). Trifluoroacetic acid (7.0 mL) was mixed with $CHCl_3$ (63 mL) to give a 10% solution. The TFA solution was added dropwise to the porphyrin solution and the reaction mixture was stirred at room temperature for 15 minutes. After the completion of the reaction, the mixture was passed immediately through a short plug of silica gel (1% pyridine in $CHCl_3$) and the solvents were removed. Recrystallization by layer addition (CH_2Cl_2/methanol) gave the product as a dark blue-green solid (1.76 g, 96%).

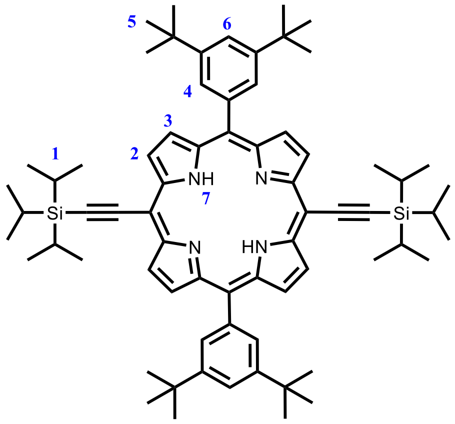

Crystals of **P1$_{2H}$** suitable for X-ray diffraction were obtained by layer addition of methanol to a solution of the porphyrin in DCM.

^{1}H NMR (400 MHz, $CDCl_3$, 298 K): δ_H (ppm) 9.65 (4H, d, J = 4.7 Hz, H2), 8.87 (4H, d, J = 4.7 Hz, H3), 8.02 (4H, d, J = 1.8 Hz, H4), 7.81 (2H, t, J = 1.8 Hz, H6), 1.55 (42H, s, H5), 1.39-1.42 (36H, m, H1), -2.10 (2H, s, H7).

MALDI-TOF: m/z = 1049 ($C_{70}H_{94}N_4Si_2$, M^+ requires 1047).

λ_{max} ($CHCl_3$) / nm log(ε): 439 (5.65), 448 (5.41), 584 (4.14), 622 (4.41), 637 (4.60).

P1$_{Gd}$

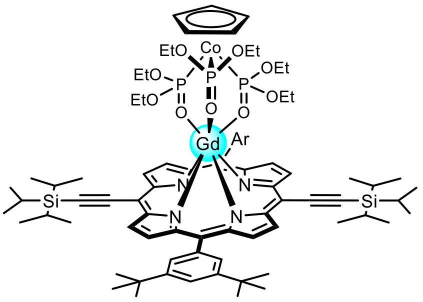

In a dry Schlenk flask, free-base porphyrin (**P1$_{2H}$**) (50 mg, 47.7 µmol), anhydrous $GdCl_3$ (126 mg, 0.47 mmol), diphenyl ether (1.0 g) and imidazole (1.0 g) were heated to 220 °C for 3 h. The mixture was allowed to cool to room temperature after which $CHCl_3$ (25 mL) was added and the mixture was washed with water to remove the imidazole. The $CHCl_3$ layer was dried over magnesium sulfate and the solvent was removed. The diphenyl ether was then removed by vacuum distillation utilizing a Hickmann apparatus. The residue was dissolved in $CHCl_3$ (5 mL), **NaL_{OEt}** (26.8 mg, 47.7 µmol) was added and the mixture was stirred at room temperature for 30 min. The reaction mixture was passed through a short plug of silica gel ($CHCl_3$). Recrystallization by layer addition (CH_2Cl_2/methanol) gave the product as a dark blue-green solid (51.7 mg, 62%).

Crystals of **P1$_{Gy}$** suitable for X-ray diffraction were obtained by layer addition of methanol to a solution of the porphyrin in chlorobenzene.

MALDI-TOF: m/z = 1739 ($C_{87}H_{127}CoGdN_4O_9P_3Si_2$, M^+ requires 1738).

λ_{max} ($CHCl_3$) / nm log(ε): 443 (5.67), 453 (5.45), 588 (4.17), 624 (4.29), 639 (4.77).

P1'$_{Gd}$

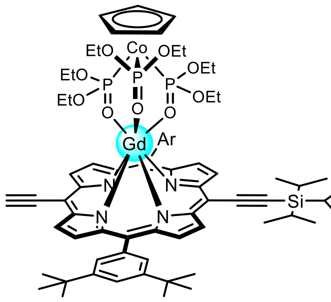

P1$_{Gd}$ (108 mg, 0.22 mmol) was dissolved in $CHCl_3$ (2 mL) and CH_2Cl_2 (8 mL). Tetra-*n*-butylammonium fluoride (1.0 mL, 1.0 M solution in THF, 1.0 mmol) was added to the stirred solution in small portions. The progress of the reaction was monitored by TLC (petrol ether:ethyl acetate:pyridine = 10:1:1) until an optimal mixture was reached (90

min). The mixture was passed through a short plug of silica gel ($CHCl_3$). The reaction mixture was purified by silica gel chromatography (petrol ether:$CHCl_3$ = 1:1 → 0:1). Recrystallization by layer addition (CH_2Cl_2/methanol) gave the product as a dark blue-green solid (18.4 mg, 19%).

MALDI-TOF: m/z = 1582 ($C_{78}H_{107}CoGdN_4O_9P_3Si$, M^+ requires 1582).

λ_{max} **($CHCl_3$) / nm log(ε)**: 442 (5.70), 453 (5.47), 587 (4.19), 624 (4.31), 640 (4.78).

$P2_{Gd2}$

$P1'_{Gd}$ (24.4 mg, 15.4 μmol) was dissolved in toluene (1 mL). A catalyst solution was prepared by dissolving $Pd(PPh_3)_2Cl_2$ (1.1 mg, 1.5 μmol), CuI (2.9 mg, 15.4 μmol) and 1,4-benzoquinone (2.2 mg, 20.5 μmol) in toluene (0.86 mL) and *i*-Pr_2NH (0.14 mL). The catalyst solution was added to the reaction mixture. The reaction mixture was stirred at room temperature for 30 min and passed through a short silica gel column ($CHCl_3$). The reaction mixture was purified by silica gel chromatography (petrol ether:$CHCl_3$ = 1:1 → 0:1). Recrystallization by layer addition (CH_2Cl_2/methanol) gave the product as a dark blue-green solid (17.4 mg, 71%).

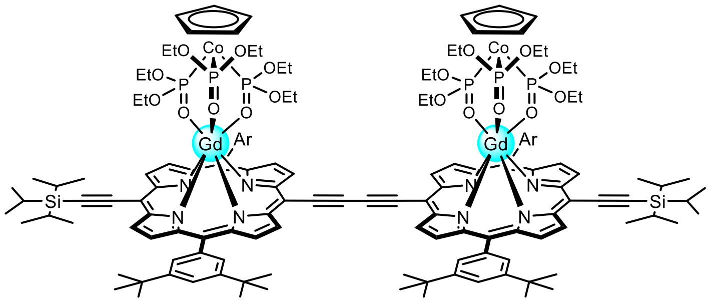

Crystals of **$P2_{Gd2}$** suitable for X-ray diffraction were obtained by dissolving the dimer in a one-to-one mixture of DCM and ethanol. Slow evaporation of the DCM allowed the dimer to slowly crystalize over time.

MALDI-TOF: m/z = 3162 ($C_{156}H_{212}Co_2Gd_2N_8O_{18}P_6Si_2$, M^+ requires 3161).

λ_{max} **($CHCl_3$) / nm log(ε)**: 435 (5.28), 460 (5.55), 496 (5.34), 586 (4.35), 667 (4.88), 733 (5.10).

$P1_{Dy}$

In a dry Schlenk flask, free-base porphyrin (**$P1_{2H}$**) (200 mg, 0.19 mmol), anhydrous $DyCl_3$ (513 mg, 1.91 mmol), diphenyl ether (4.0 g) and imidazole (4.0 g) were heated to 220 °C for 3 h. The mixture was allowed to cool to room temperature after which $CHCl_3$ (50 mL) was added and the mixture was washed with water to remove the imidazole. The $CHCl_3$ layer was dried over magnesium sulfate and the solvent was removed. The diphenyl ether was then removed by

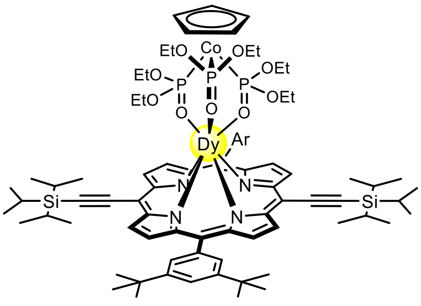

vacuum distillation utilizing a Hickmann apparatus. The residue was dissolved in $CHCl_3$ (20 mL), **NaL_{OEt}** (104 mg, 0.19 mmol) was added and the mixture was stirred at room temperature for 30 min. The reaction mixture was passed through a short plug of silica gel ($CHCl_3$). Recrystallization by layer addition (CH_2Cl_2/methanol) gave the product as a dark blue-green solid (235 mg, 71%).

Crystals of **$P1_{Dy}$** suitable for X-ray diffraction were obtained by layer addition of methanol to a solution of the porphyrin in chlorobenzene.

MALDI-TOF: m/z = 1744 ($C_{87}H_{127}CoN_4O_9P_3Si_2Dy$, M^+ requires 1744).
λ_{max} ($CHCl_3$) / nm log(ε): 443 (5.68), 453 (5.47), 587 (4.20), 624 (4.33), 637 (4.78).

$P1'_{Dy}$

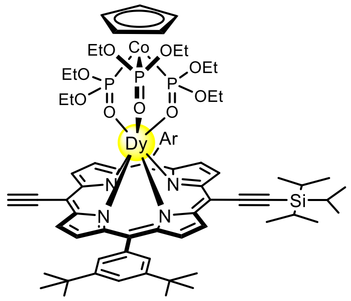

$P1_{Dy}$ (230 mg, 0.13 mmol) was dissolved in $CHCl_3$ (2 mL) and CH_2Cl_2 (10 mL). Tetra-*n*-butylammonium fluoride (1.0 mL, 1.0 M solution in THF, 1.0 mmol) was added to the stirred solution in small portions. The progress of the reaction was monitored by TLC (petrol ether:ethyl acetate:pyridine = 10:1:1) until an optimal mixture was reached (60 min). The mixture was passed through a short plug of silica gel ($CHCl_3$). The reaction mixture was purified by silica gel chromatography (petrol ether:$CHCl_3$ = 1:1 → 0:1). Recrystallization by layer addition (CH_2Cl_2/methanol) gave the product as a dark blue-green solid (52.7 mg, 25%).

MALDI-TOF: m/z = 1588 ($C_{78}H_{107}CoDyN_4O_9P_3Si$, M^+ requires 1588).
λ_{max} ($CHCl_3$) / nm log(ε): 441 (5.54), 450 (5.42), 583 (4.16), 632 (4.59).

$P2_{Dy2}$

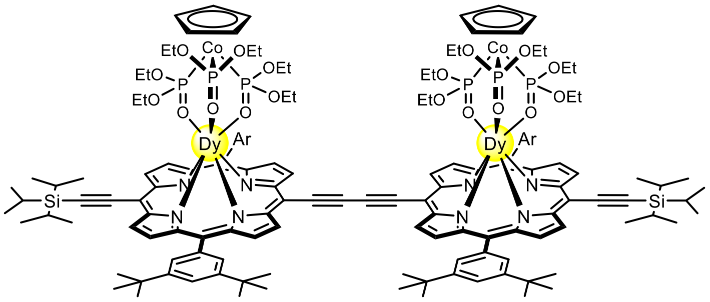

$P1'_{Dy}$ (52.7 mg, 33.3 μmol) was dissolved in toluene (2 mL). A catalyst solution was prepared by dissolving $Pd(PPh_3)_2Cl_2$ (2.3 mg, 3.3 μmol), CuI (6.3 mg, 33.3 μmol) and 1,4-benzoquinone (4.8 mg, 44.3 μmol) in toluene (2.1 mL) and *i*-Pr_2NH (0.35 mL). The catalyst solution was added to the reaction mixture. The reaction mixture was stirred at room temperature for 1 h and passed through a short silica gel column ($CHCl_3$). The reaction mixture was purified by silica gel chromatography (petrol ether:$CHCl_3$ = 1:1 → 0:1).

Recrystallization by layer addition (CH_2Cl_2/methanol) gave the product as a dark blue-green solid (25.1 mg, 48%).

Crystals of **P2$_{Dy2}$** suitable for X-ray diffraction were obtained by dissolving the dimer in a one-to-one mixture of DCM and ethanol. Slow evaporation of the DCM allowed the dimer to slowly crystalize over time.

MALDI-TOF: m/z = 3172 ($C_{156}H_{212}Co_2Dy_2N_8O_{18}P_6Si_2$, M^+ requires 3172).
λ_{max} ($CHCl_3$) / nm log(ε): 435 (5.27), 460 (5.55), 496 (5.30), 584 (4.32), 666 (4.87), 731 (5.07).

P1$_{Tb}$

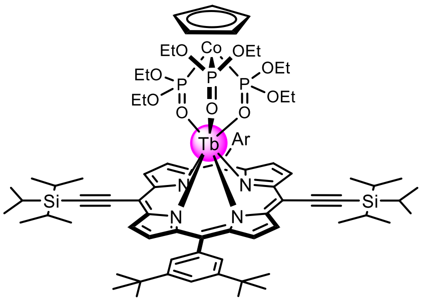

In a dry Schlenk flask, free-base porphyrin (**P1$_{2H}$**) (200 mg, 0.19 mmol), $TbCl_3{\cdot}6H_2O$ (710 mg, 1.91 mmol), diphenyl ether (4.0 g) and imidazole (4.0 g) were heated to 190 °C for 2 h. The mixture was allowed to cool to room temperature after which $CHCl_3$ (50 mL) was added and the mixture was washed with water to remove the imidazole. The $CHCl_3$ layer was dried over sodium sulfate and the solvent was removed. The diphenyl ether was then removed by vacuum distillation utilizing a Hickmann apparatus. The residue was dissolved in $CHCl_3$ (5 mL), **NaL$_{OEt}$** (109 mg, 0.19 mmol) was added and the mixture was stirred at room temperature for 17 h. The reaction mixture was concentrated and purified by silica gel column chromatography (petroleum ether : ethyl acetate : pyridine = 20 : 1 : 1). Recrystallization by layer addition (CH_2Cl_2/methanol) gave the product as a dark blue-green solid (203 mg, 61%).

Crystals of **P1$_{Tb}$** suitable for X-ray diffraction were obtained by layer addition of methanol to a solution of the porphyrin in chlorobenzene.

MALDI-TOF: m/z = 1740 ($C_{87}H_{127}CoN_4O_9P_3Si_2Tb$, M^+ requires 1740).
λ_{max} ($CHCl_3$) / nm log(ε): 440 (5.71), 579 (4.24), 626 (4.36).

P1'$_{Tb}$

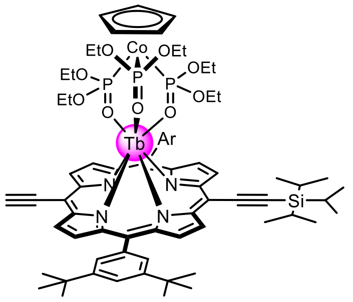

P1$_{Tb}$ (143 mg, 82 μmol) was dissolved in $CHCl_3$ (1 mL) and CH_2Cl_2 (5 mL). Tetra-*n*-butylammonium fluoride (0.12 mL, 1.0 M solution in THF, 0.12 mmol) was added to the stirred solution in small portions. The progress of the reaction was monitored by TLC (petrol ether:ethyl acetate:pyridine = 10:1:1) until an optimal mixture was reached (80

min). The mixture was passed through a short plug of silica gel ($CHCl_3$). The reaction mixture was purified by silica gel chromatography (petrol ether:ethyl acetate:pyridine = 20:1:1→ 10:1:1). Recrystallization by layer addition (CH_2Cl_2/methanol) gave the product as a dark blue-green solid (65.1 mg, 50%).

MALDI-TOF: m/z = 1584 ($C_{78}H_{107}CoN_4O_9P_3SiTb$, M^+ requires 1583).

λ_{max} ($CHCl_3$) / nm log(ε): 442 (5.76), 580 (4.27), 628 (4.41).

$P1_{Zn}$*

tris-(Dibenzylideneacetone)-di-palladium(0) (50.4 mg, 55 mmol), copper(I) iodide (21.0 mg, 0.11 mmol), triphenylphosphine (28.9 mg, 0.11 mmol) and dibromoporphyrin (500 mg, 0.55 mmol) were placed in a dried two-neck roundbottom flask under argon. Toluene (35 mL), *i*-Pr_2NH (19 mL) and pyridine (1 mL) were added and the reaction mixture was deoxygenated.

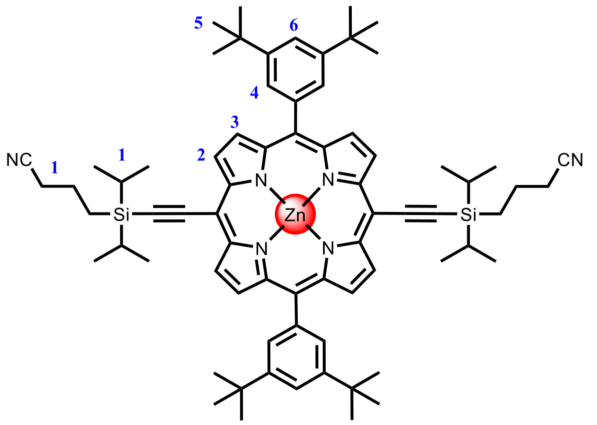

Cyanopropyldiisopropylsilyl acetylene (343 mg, 1.65 mmol) was added by syringe. The reaction mixture was stirred at 50 °C for 2 h, and monitored by TLC. The solvents were removed and the residue was passed through a short silica gel plug using CH_3Cl containing 1% pyridine. The reaction mixture was purified by silica gel chromatography (petrol ether:ethyl acetate:pyridine = 10:1:1). Recrystallization by layer addition (CH_2Cl_2/methanol) gave the product as a dark blue-green solid (201 mg, 31%).

Crystals of **$P1_{Zn}$*** suitable for X-ray diffraction were obtained by layer addition of methanol to a solution of the porphyrin in DCM.

^{1}H NMR (400 MHz, $CDCl_3$, 298 K): δ_H (ppm) 9.64 (4H, d, *J* = 4.6 Hz, H2), 8.92 (4H, d, *J* = 4.6 Hz, H3), 7.99 (4H, d, *J* = 1.8 Hz, H4), 7.80 (2H, t, *J* = 1.8 Hz, H6), 1.55 (36H, s, H5), 2.57-0.92 (40H, m, H1).

MALDI-TOF: *m/z* = 1161 ($C_{72}H_{90}N_6Si_2Zn$, M^+ requires 1161).

λ_{max} ($CHCl_3$) / nm log(ε): 439 (5.65), 448 (5.41), 584 (4.14), 622 (4.41), 637 (4.60).

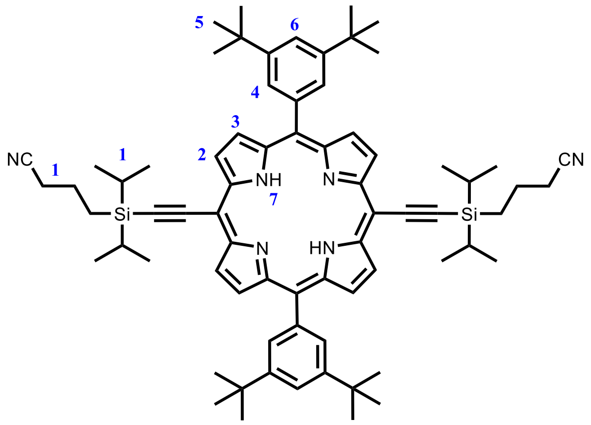

P1$_{2H}$*

P1$_{Zn}$* (201 mg, 0.172 mmol) was dissolved in $CHCl_3$ (33 mL). Trifluoroacetic acid (0.68 mL) was mixed with $CHCl_3$ (6 mL) to give a 10% solution. The TFA solution was added dropwise to the porphyrin solution and the reaction mixture was stirred at room temperature for 15 minutes. The reaction mixture was passed through a short plug of silica gel ($CHCl_3$). Recrystallization by layer addition (CH_2Cl_2/methanol) gave the product as a dark blue-green solid (147 mg, 78%).

Crystals of **P1$_{2H}$** suitable for X-ray diffraction were obtained by layer addition of methanol to a solution of the porphyrin in DCM.

^{1}H NMR (400 MHz, $CDCl_3$, 298 K): δ_H (ppm) 9.61 (4H, d, J = 4.7 Hz, H2), 8.89 (4H, d, J = 4.7 Hz, H3), 8.02 (4H, d, J = 1.8 Hz, H4), 7.82 (2H, t, J = 1.8 Hz, H6), 1.55 (42H, s, H5), 2.62-0.76 (40H, m, H1), -2.12 (2H, s, H7).

λ_{max} ($CHCl_3$) / nm log(ε): 434 (5.65), 441 (5.55), 510 (3.88), 544 (4.18), 585 (4.78), 622 (3.90), 680 (4.43).

MALDI-TOF: m/z = 1097 ($C_{72}H_{92}N_6Si_2$, M^+ requires 1098).

P1$_{Dy}$*

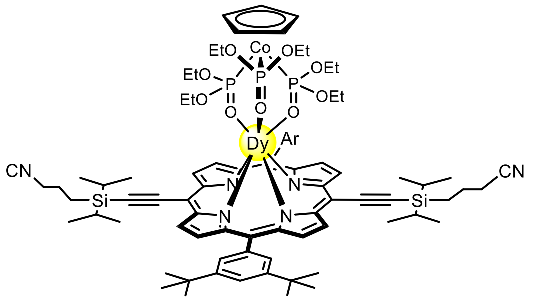

In a dry Schlenk flask, free-base porphyrin (**P1$_{2H}$***) (100 mg, 0.09 mmol), anhydrous $DyCl_3$ (343 mg, 0.91 mmol), diphenyl ether (2.0 g) and imidazole (2.0 g) were heated to 210 °C for 3 h. The mixture was allowed to cool to room temperature after which $CHCl_3$ (100 mL) was added and the mixture was washed with water to remove the imidazole. The $CHCl_3$ layer was dried over sodium sulfate and the solvent was removed. The diphenyl ether was then removed by vacuum distillation utilizing a Hickmann apparatus. The residue was dissolved in $CHCl_3$ (2.5 mL), **NaL$_{OEt}$** (51 mg, 0.09 mmol) was added and the mixture was stirred at room temperature for 16 h. The reaction mixture was passed through a short plug of silica gel ($CHCl_3$). Recrystallization by layer addition (CH_2Cl_2/methanol) gave the product as a dark blue-green solid (21.4 mg, 13%).

Crystals of **P1$_{Dy}$*** suitable for X-ray diffraction were obtained by layer addition of methanol to a solution of the porphyrin in chlorobenzene.

MALDI-TOF: m/z = 1794 ($C_{89}H_{125}CoDyN_6O_9P_3Si_2$, M^+ requires 1794).

λ_{max} **($CHCl_3$) / nm log(ε)**: 442 (5.67), 452 (5.43), 587 (4.15), 624 (4.33), 638 (4.72).

P1'$_{Dy}$*

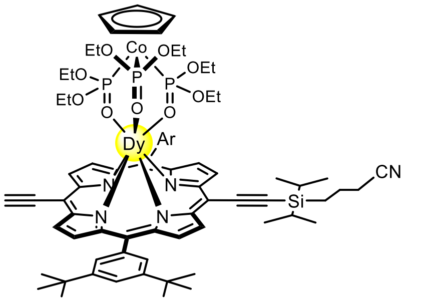

P1$_{Dy}$* (21.4 mg, 13 μmol) was dissolved in CH_2Cl_2 (1 mL) and $CHCl_3$ (1 mL). Tetra-*n*-butylammonium fluoride (18 μL, 1.0 M solution in THF, 18 μmol) was added to the stirred solution in small portions. The progress of the reaction was monitored by TLC (petrol ether:ethyl acetate:pyridine = 10:1:1) until an optimal mixture was reached (60 min). The mixture was passed through a short plug of silica gel ($CHCl_3$). The reaction mixture was purified by silica gel chromatography (petrol ether:ethyl acetate:pyridine = 20:1:1 → 10:1:1). Recrystallization by layer addition (CH_2Cl_2/methanol) gave the product as a dark blue-green solid (8.4 mg, 44%).

MALDI-TOF: m/z = 1613 ($C_{79}H_{106}CoDyN_5O_9P_3Si$, M^+ requires 1613).

λ_{max} **($CHCl_3$) / nm log(ε)**: 444 (5.68), 453 (5.41), 588 (4.18), 626 (4.35), 639 (4.73).

P2$_{Tb2}$ and P2$_{Dy-Tb}$

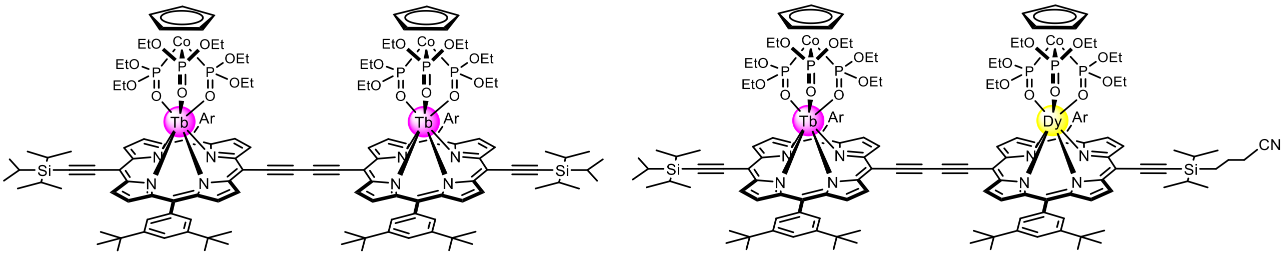

P1'$_{Dy}$* (8.4 mg, 5.2 μmol) and **P1'$_{Tb}$** (65.1 mg, 41.9 μmol) was dissolved in toluene (2.5 mL). A catalyst solution was prepared by dissolving $Pd(PPh_3)_2Cl_2$ (3.3 mg, 4.7 μmol), CuI (9.0 mg, 47.1 μmol) and 1,4-benzoquinone (6.8 mg, 62.8 μmol) in toluene (3 mL) and *i*-Pr_2NH (0.5 mL). The catalyst solution was added to the reaction mixture. The reaction mixture was stirred at room temperature for 1 h and passed through a short silica gel column ($CHCl_3$). The two formed dimers were separated by silica gel chromatography (cyclohexane:CH_2Cl_2 = 1:1 → 0:1). **P2Tb$_2$** was recrystallised by layer addition of methanol to a solution in CH_2Cl_2, to give the product as a dark blue-green solid (21.4 mg, 33%, yield calculated from P1'Tb). **P2Dy-Tb** was also recrystallised from CH_2Cl_2/methanol to give the product as a dark blue solid (4.1 mg, 25%, yield calculated from P1'Dy*). The reaction also generated a small amount of **P2Dy$_{2*}$** but this product was not isolated.

MALDI-TOF P2$_{Tb2}$: m/z = 3165 ($C_{156}H_{212}Co_2N_8O_{18}P_6Si_2Tb_2$, M^+ requires 3165).

MALDI-TOF P2$_{Dy-Tb}$: m/z = 3194 ($C_{157}H_{212}Co_2DyN_9O_{18}P_6Si_2Tb$, M^+ requires 3193).

λ_{max} **P2$_{Tb2}$ (CHCl$_3$) / nm log(ε)**: 435 (5.25), 460 (5.55), 496 (5.30), 584 (4.33), 666 (4.99), 731 (5.14).

λ_{max} **P2$_{Dy\text{-}Tb}$ (CHCl$_3$) / nm log(ε)**: 435 (5.25), 460 (5.55), 496 (5.29), 584 (4.34), 666 (4.86), 731 (5.06).

P1$_Y$

A diamagnetic yttrium analogue was prepared to investigate the structure of a capped porphyrin by ^{1}H-NMR spectroscopy.

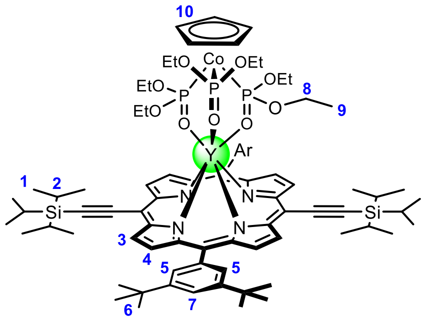

In a dry Schlenk flask, free-base porphyrin (**P1$_{2H}$**) (100 mg, 0.10 mmol), $YCl_3{\cdot}6H_2O$ (289 mg, 0.95 mmol), diphenyl ether (2.0 g) and imidazole (2.0 g) were heated to 220 °C for 2 h. The mixture was allowed to cool to room temperature after which CHCl$_3$ (50 mL) was added and the mixture was washed with water to remove the imidazole. The CHCl$_3$ layer was dried over sodium sulfate and the solvent was removed. The diphenyl ether was then removed by vacuum distillation utilizing a Hickmann apparatus. The residue was dissolved in CHCl$_3$ (2.5 mL), **NaL$_{OEt}$** (51 mg, 0.10 mmol) was added and the mixture was stirred at room temperature for 1 h. The reaction mixture was concentrated and purified by silica gel column chromatography (petroleum ether : ethyl acetate : pyridine = 20 : 1 : 1). Recrystallization by layer addition (CH$_2$Cl$_2$/methanol) gave the product as a dark blue-green solid (111 mg, 70%).

Crystals of **P1$_Y$** suitable for X-ray diffraction were obtained by layer addition of methanol to a solution of the porphyrin in chlorobenzene.

^{1}H NMR (400 MHz, CDCl$_3$, 298 K): δ_H (ppm) 9.57 (4H, d, *J* = 4.5 Hz, H3), 8.72 (4H, d, *J* = 4.5 Hz, H4), 8.28 (2H, t, *J* = 1.6 Hz, H5), 7.75 (2H, t, *J* = 1.8 Hz, H7), 7.63 (2H, t, *J* = 1.5 Hz, H5), 4.11 (5H, s, H10), 2.64 (6H, m, H8), 2.54 (6H, m, H8), 1.63 (18H, s, H1), 1.43 (42H, s, H2 and H6), 0.77 (18H, t, *J* = 7.1 Hz, H9).

λ_{max} **(CHCl$_3$) / nm log(ε)**: 443 (5.67), 453 (5.43), 588 (4.17), 624 (4.29), 639 (4.78).

1.3 Spectra Confirming Identity of New Compounds

P1$_{Gd}$

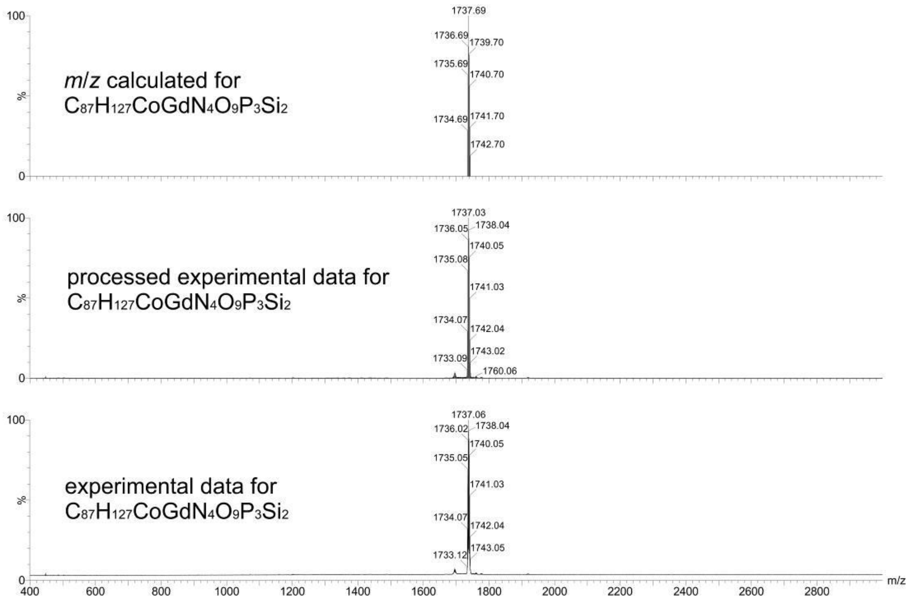

Figure S2. The full MALDI-MS spectrum of **P1$_{Gd}$** (*m/z* = 1737 ($C_{87}H_{127}CoGdN_4O_9P_3Si_2$, M^+ requires 1738), matrix: dithranol).

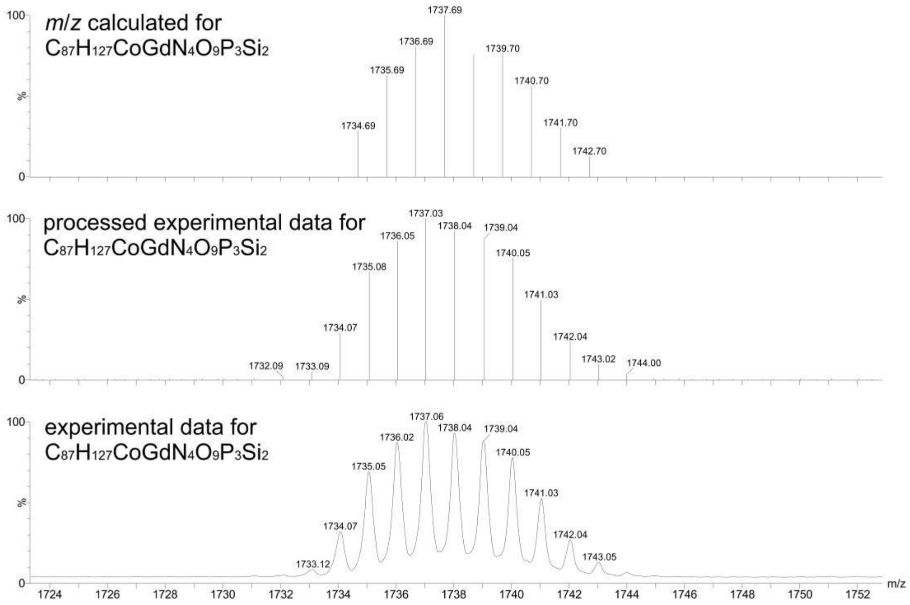

Figure S3. Zoom-in of the MALDI-MS spectrum of **P1$_{Gd}$** (*m/z* = 1737 ($C_{87}H_{127}CoGdN_4O_9P_3Si_2$, M^+ requires 1738), matrix: dithranol).

P2Gd2

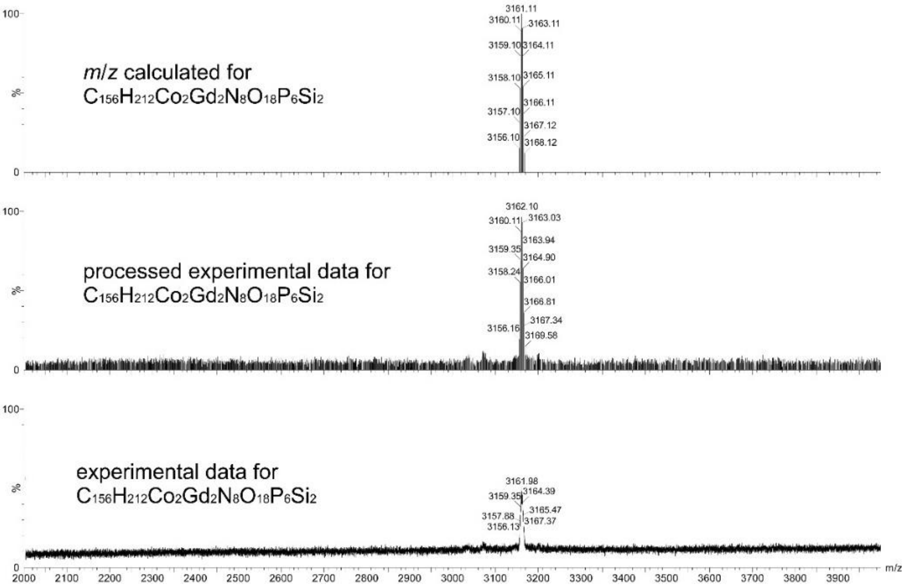

Figure S4. The full MALDI-MS spectrum of **P2Gd2** (m/z = 3162 ($C_{156}H_{212}Co_2Gd_2N_8O_{18}P_6Si_2$, M^+ requires 3161), matrix: dithranol).

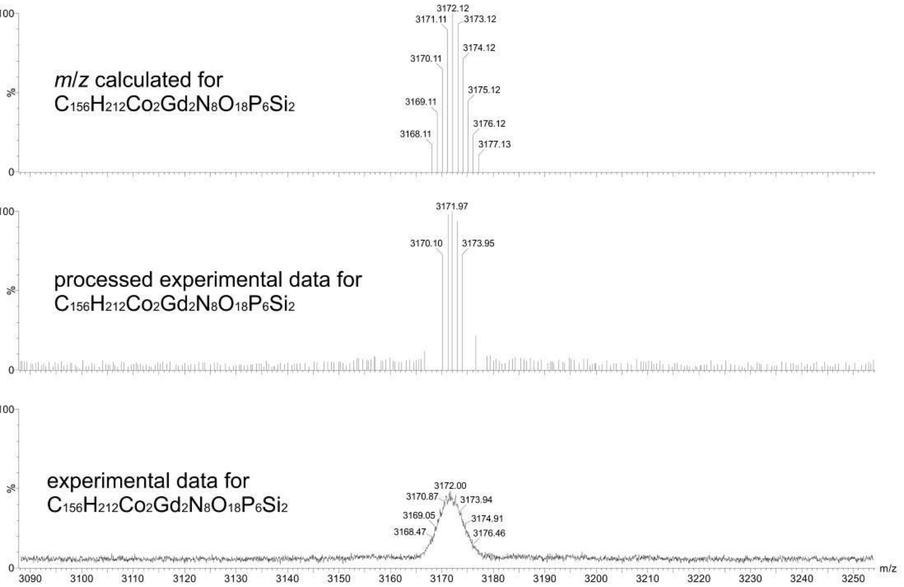

Figure S5. Zoom-in of the MALDI-MS spectrum of **P2Gd2** (m/z = 3162 ($C_{156}H_{212}Co_2Gd_2N_8O_{18}P_6Si_2$, M^+ requires 3161), matrix: dithranol).

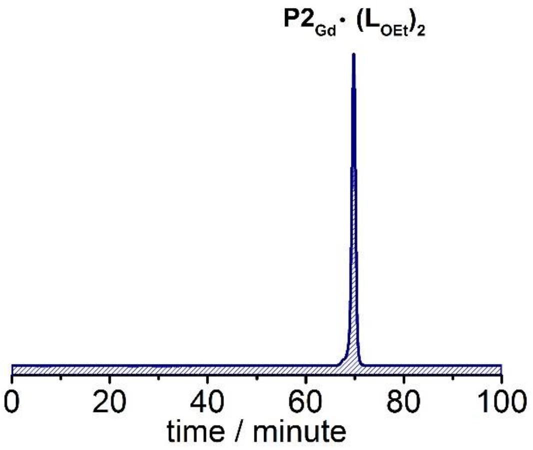

Figure S6. Analytical GPC trace (PLGel columns, toluene + 1% pyridine, detection at 731 nm) of **$P2_{Gd2}$**.

$P1_{Dy}$

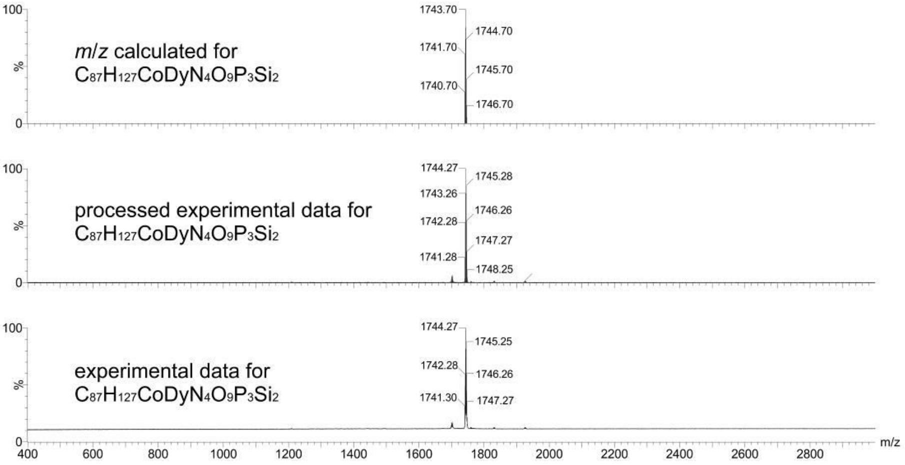

Figure S7. The full MALDI-MS spectrum of **$P1_{Dy}$** (m/z = 1744 ($C_{87}H_{127}CoDyN_4O_9P_3Si_2$, M^+ requires 1744), matrix: dithranol).

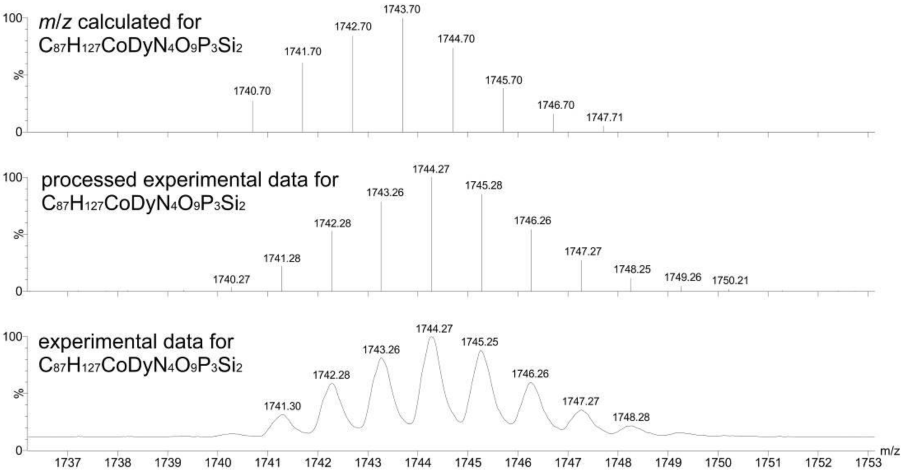

Figure S8. Zoom-in of the MALDI-MS spectrum of **$P1_{Dy}$** (m/z = 1744 ($C_{87}H_{127}CoDyN_4O_9P_3Si_2$, M^+ requires 1744), matrix: dithranol).

P2$_{Dy2}$

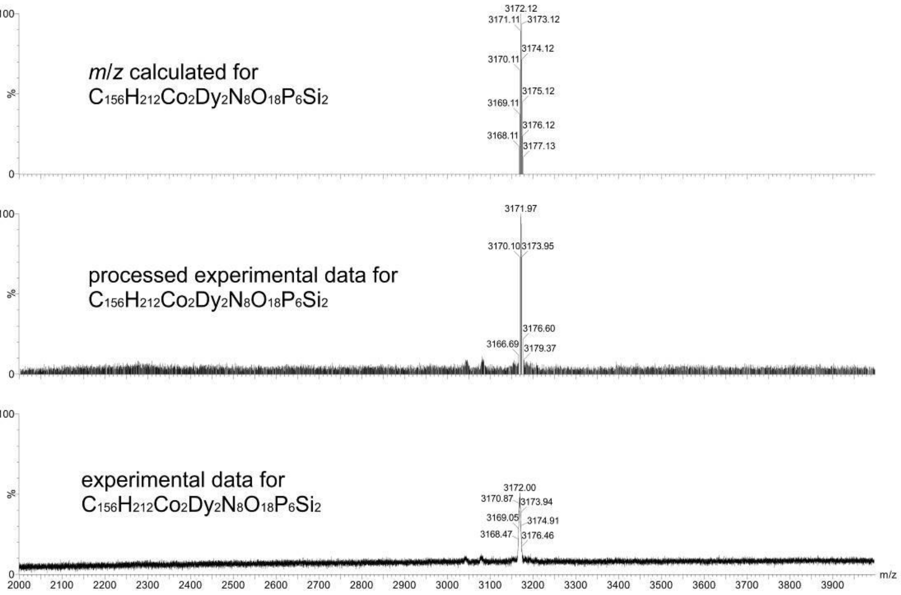

Figure S9. The full MALDI-MS spectrum of **P2$_{Dy2}$** (*m*/*z* = 3172 ($C_{156}H_{212}Co_2Dy_2N_8O_{18}P_6Si_2$, M^+ requires 3172), matrix: dithranol).

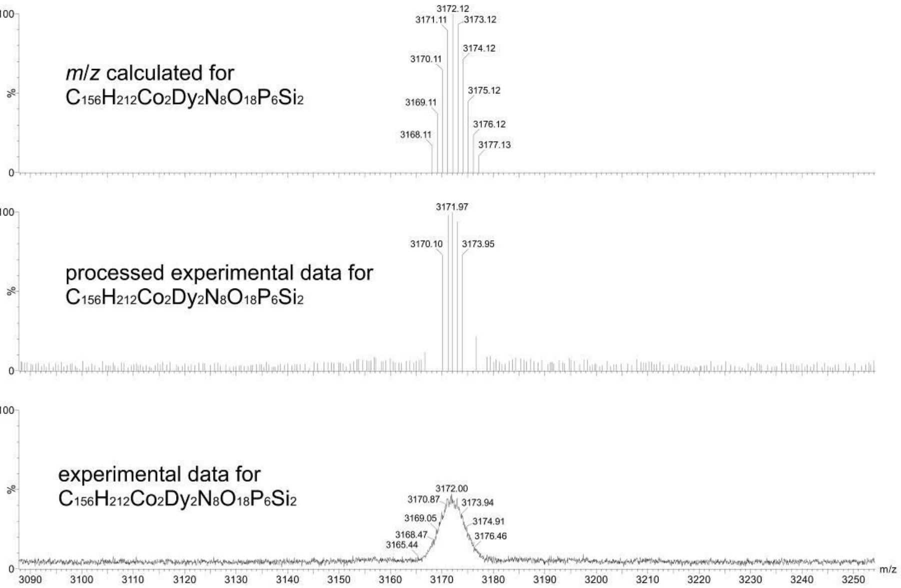

Figure S10. Zoom-in of the MALDI-MS spectrum of **P2$_{Dy2}$** (*m*/*z* = 3172 ($C_{156}H_{212}Co_2Dy_2N_8O_{18}P_6Si_2$, M^+ requires 3172), matrix: dithranol).

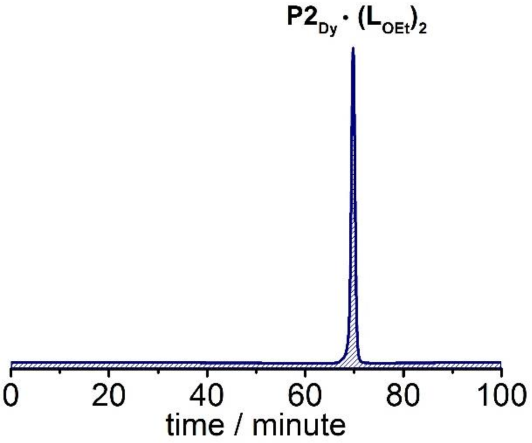

Figure S11. Analytical GPC trace (PLGel columns, toluene + 1% pyridine, detection at 731 nm) of **P2$_{Dy2}$**.

P1$_{Tb}$·(L$_{OEt}$)

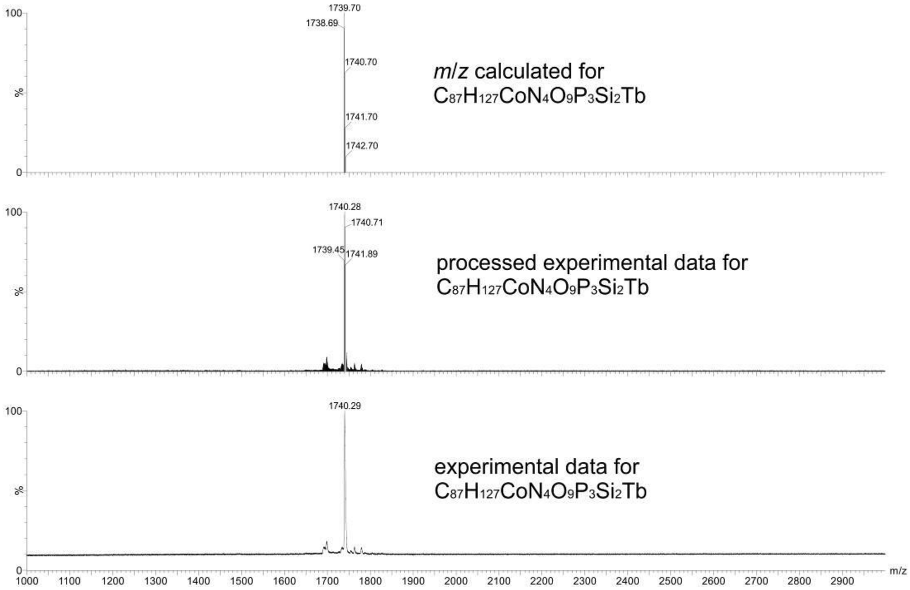

Figure 12: The full MALDI-MS spectrum of **P1$_{Tb}$** (m/z = 1740 ($C_{87}H_{127}CoN_4O_9P_3Si_2Tb$, M^+ requires 1740), matrix: dithranol).

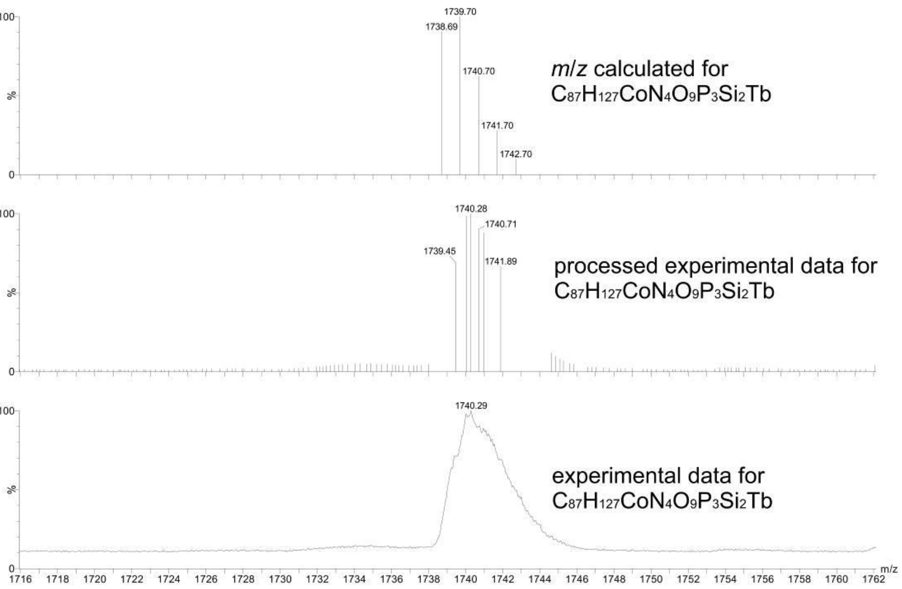

Figure S13. Zoom-in of the MALDI-MS spectrum of **P1$_{Tb}$** (m/z = 1740 ($C_{87}H_{127}CoN_4O_9P_3Si_2Tb$, M^+ requires 1740), matrix: dithranol).

P1'$_{Tb}$

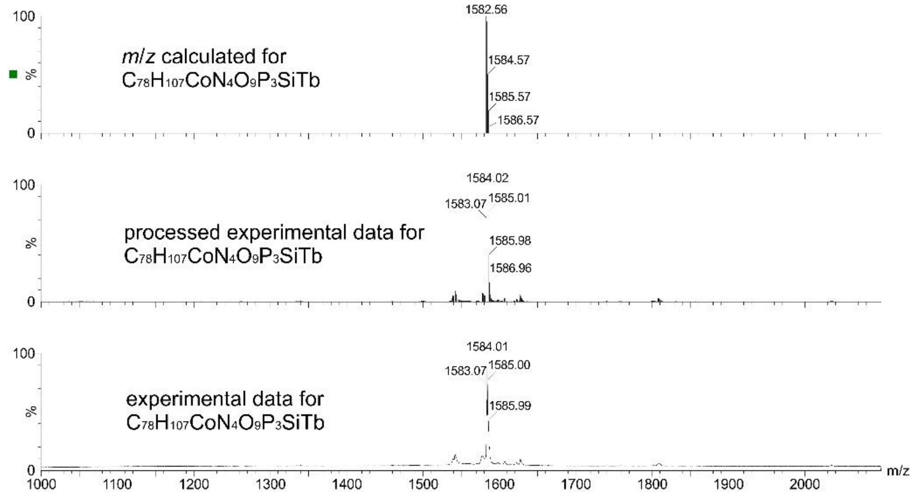

Figure S14. The full MALDI-MS spectrum of **P1'$_{Tb}$** (*m*/*z* = 1584 ($C_{78}H_{107}CoN_4O_9P_3SiTb$, M^+ requires 1583), matrix: dithranol).

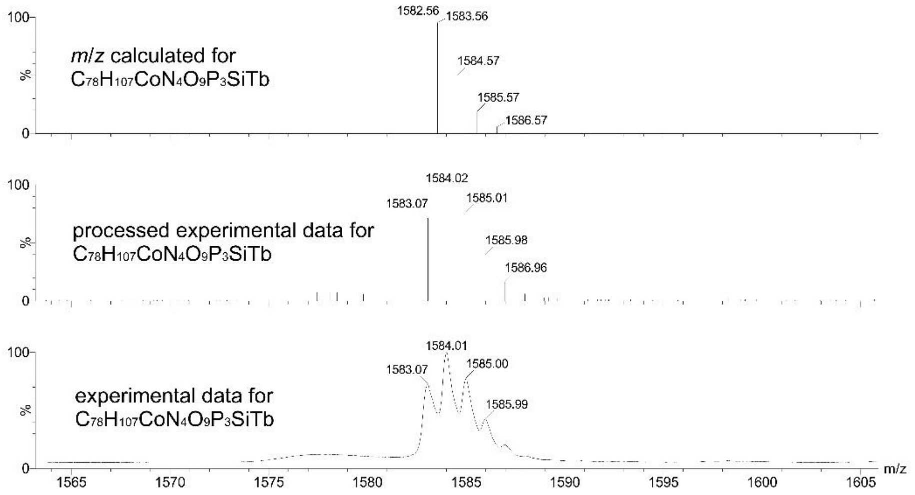

Figure S15. Zoom-in of the MALDI-MS spectrum of **P1'$_{Tb}$** (m/z = 1584 ($C_{78}H_{107}CoN_4O_9P_3SiTb$, M^+ requires 1583), matrix: dithranol).

P2$_{Tb2}$

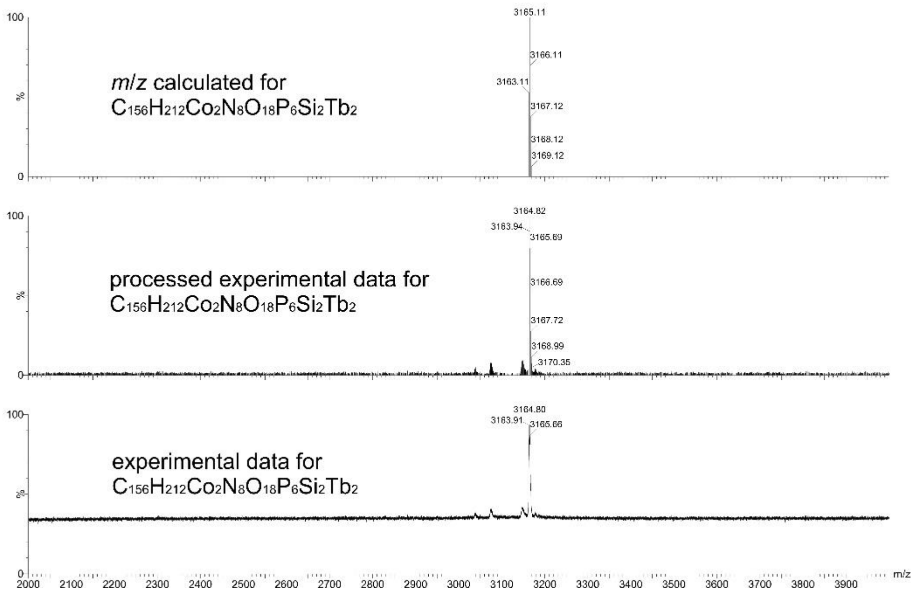

Figure S16. The full MALDI-MS spectrum of **P2$_{Tb2}$** (m/z = 3165 ($C_{156}H_{212}Co_2N_8O_{18}P_6Si_2Tb_2$, M^+ requires 3165), matrix: dithranol).

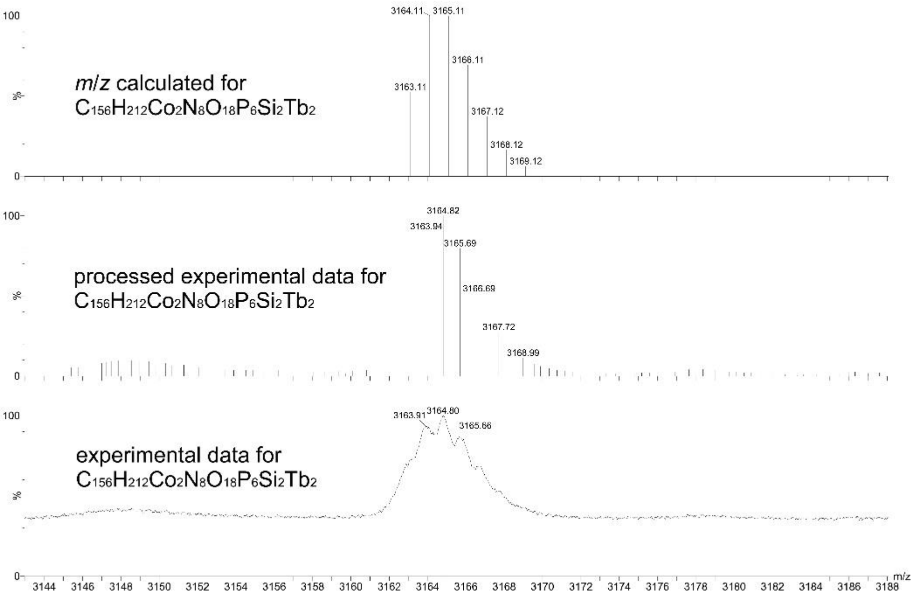

Figure S17. Zoom-in of the MALDI-MS spectrum of **P2$_{Tb2}$** (*m/z* = 3165 ($C_{156}H_{212}Co_2N_8O_{18}P_6Si_2Tb_2$, M^+ requires 3165), matrix: dithranol).

P1$_{Dy}$

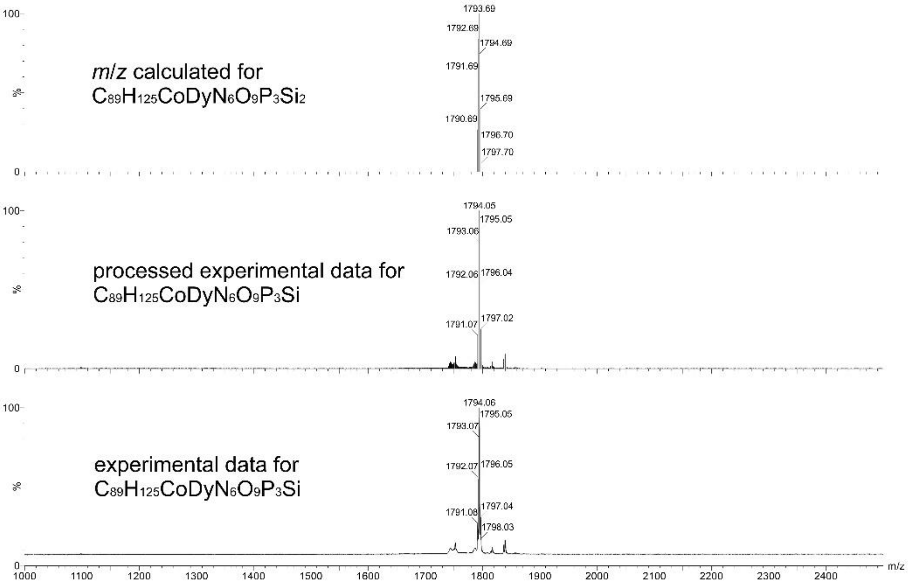

Figure S18. The full MALDI-MS spectrum of **P1$_{Dy}$** (*m/z* = 1794 ($C_{89}H_{125}CoDyN_6O_9P_3Si_2$, M^+ requires 1794), matrix: dithranol).

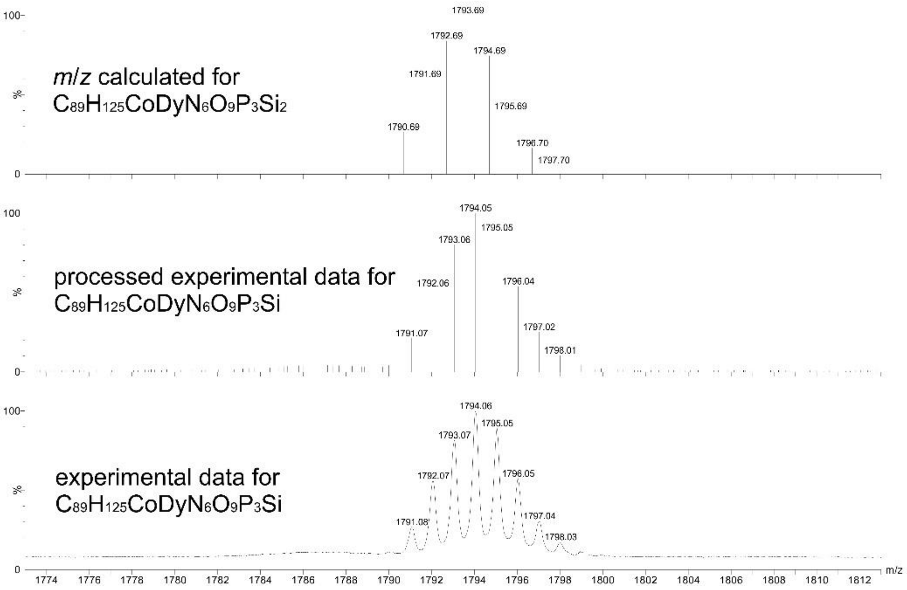

Figure S19. Zoom-in of the MALDI-MS spectrum of **$\mathbf{P1_{Dy}}$** (*m/z* = 1794 ($C_{89}H_{125}CoDyN_6O_9P_3Si_2$, M^+ requires 1794), matrix: dithranol).

$\mathbf{P1'_{Dy}}$

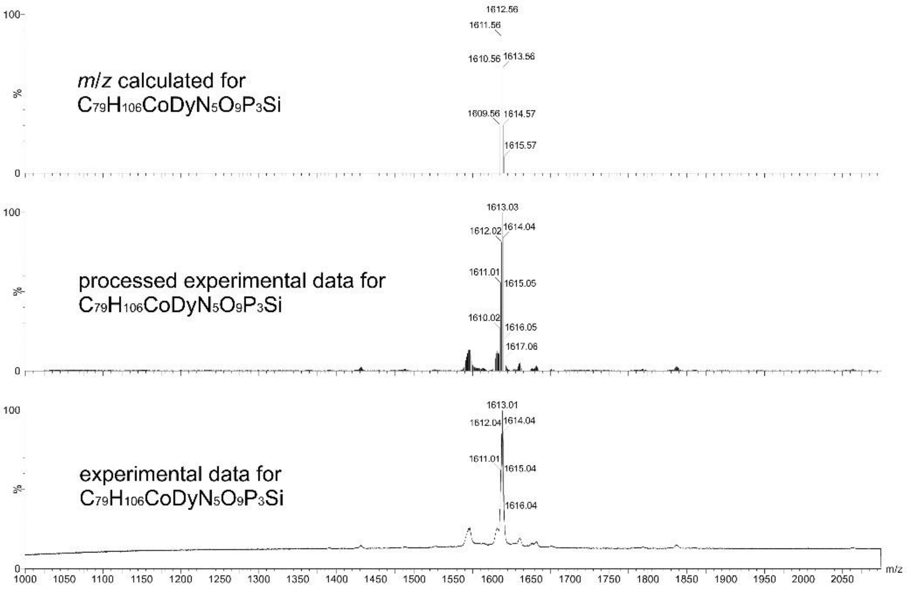

Figure S20. The full MALDI-MS spectrum of **$\mathbf{P1'_{Dy}}$** (*m/z* = 1613 ($C_{79}H_{106}CoDyN_5O_9P_3Si$, M^+ requires 1613), matrix: dithranol).

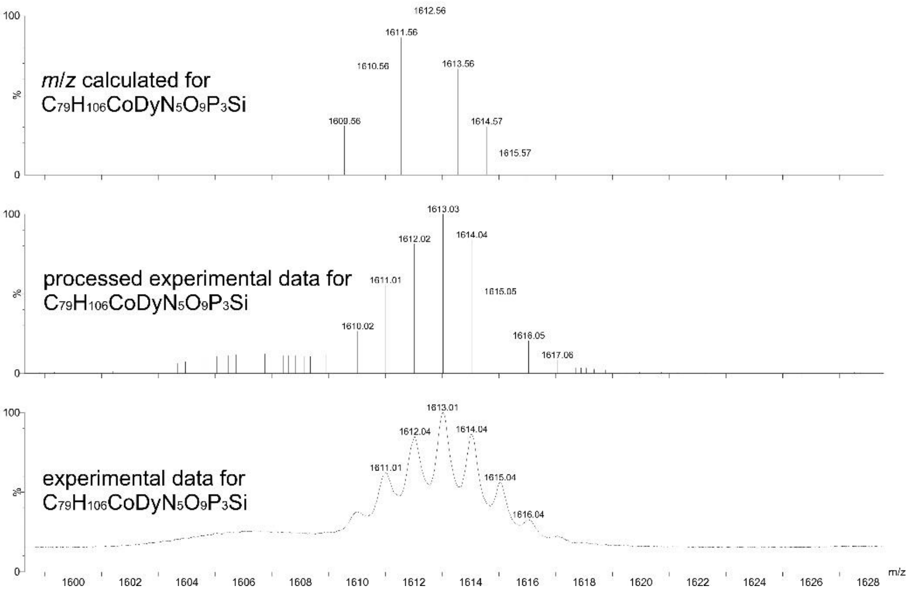

Figure S21. Zoom-in of MALDI-MS spectrum of **P1'$_{Dy}$** (m/z = 1613 ($C_{79}H_{106}CoDyN_5O_9P_3Si$, M^+ requires 1613), matrix: dithranol).

P2$_{Dy\text{-}Tb}$

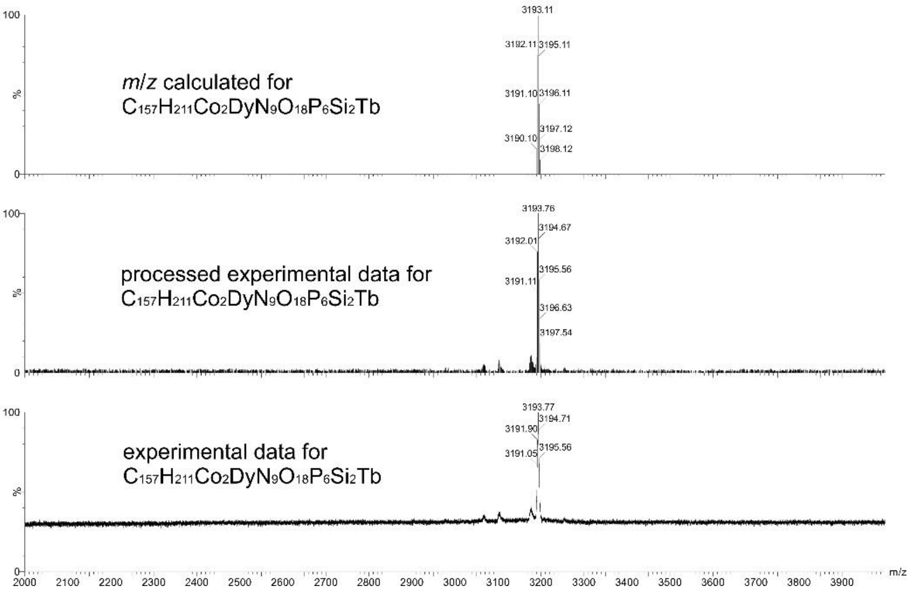

Figure S22. The full MALDI-MS spectrum of **P2$_{Dy\text{-}Tb}$** (m/z = 3194 ($C_{157}H_{211}Co_2DyN_9O_{18}P_6Si_2Tb$, M^+ requires 3193), matrix: dithranol).

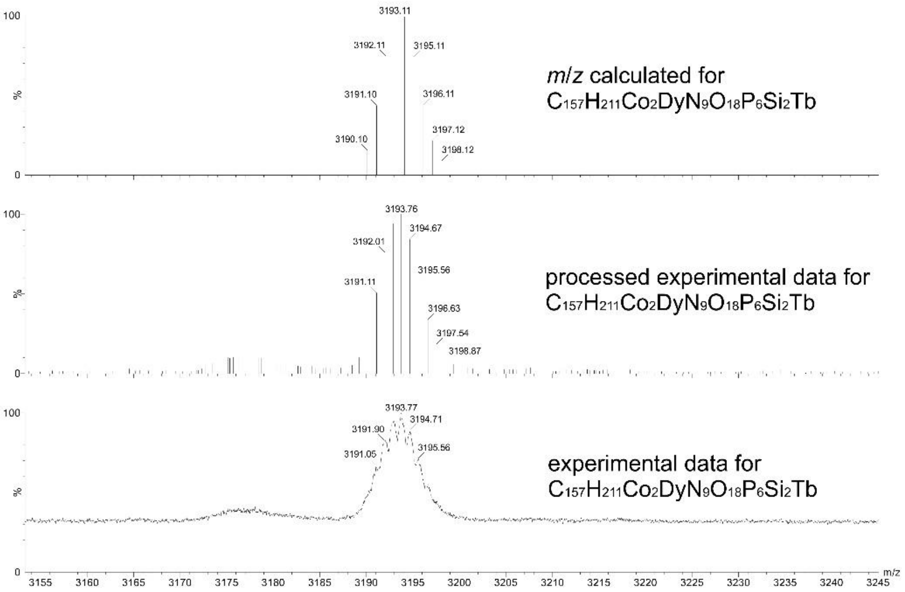

Figure S23. Zoom-in of the MALDI-MS spectrum of **P2**$_{\mathbf{Dy\text{-}Tb}}$ (*m/z* = 3194 ($C_{157}H_{211}Co_2DyN_9O_{18}P_6Si_2Tb$, M^+ requires 3193), matrix: dithranol).

1.4 UV-vis-NIR Spectroscopy

UV-vis-NIR spectra were recorded of the lanthanide monomers and dimers showing only marginal changes in the absorption spectra across the lanthanide series. The comparability of the spectra indicates the purity of the compounds.

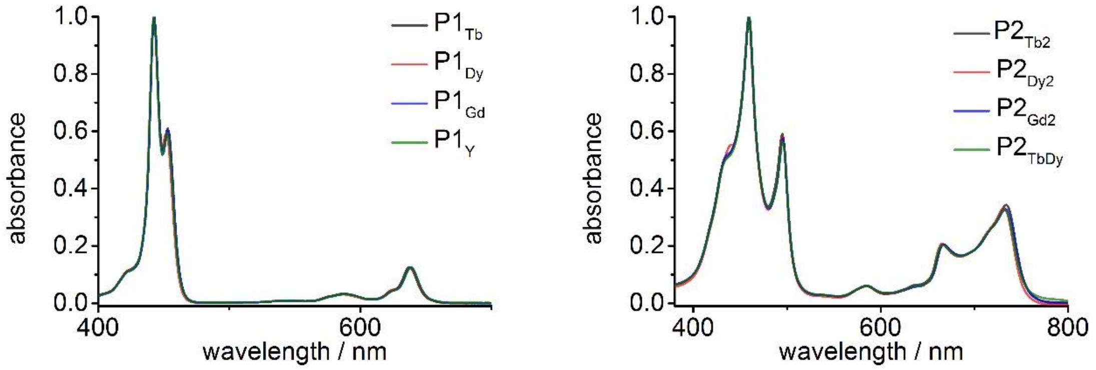

Figure S24. Normalized UV-vis-NIR spectra of **P1**$_{\mathbf{Ln}}$ and **P2**$_{\mathbf{Ln}}$ ($CHCl_3$, 298K) showing only marginal differences across the lanthanide series.

A slight red-shift in the B-band is observed when comparing a zinc porphyrin monomers and dimers with their terbium analogue.

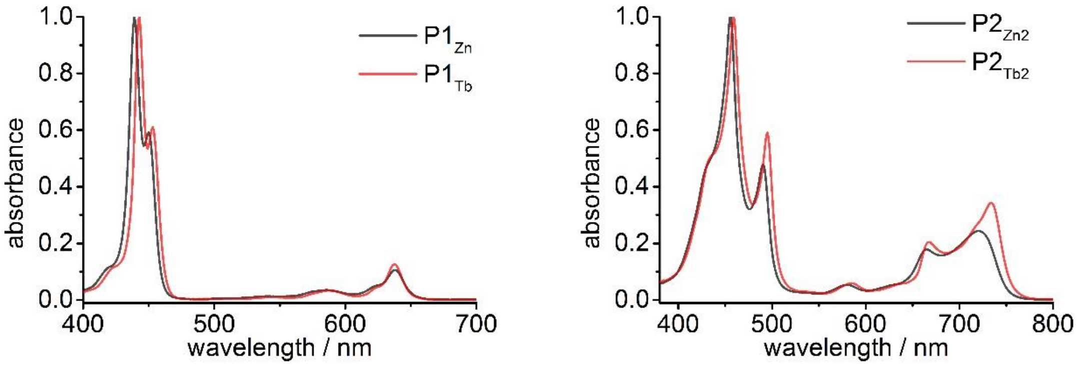

Figure S25. Normalized UV-vis-NIR spectra comparing **P1$_{Zn}$** with **P1$_{Tb}$** and **P2$_{Zn2}$** with **P2$_{Tb2}$** ($CHCl_3$, 298K).

2 X-ray Diffraction

2.1 General Experimental Details

A suitable crystal for each sample was selected, mounted on a MITIGEN holder in oil and data collected following a standard method.[7] Samples **P1'$_{Dy}$**, **P1$_{Dy}$**, **P1$_{Gd}$**, **P1*$_{2H}$**, **P1*$_{Dy}$**, **P1$_{Zn}$**, **P2$_{Dy2}$** and **P2$_{Tb2}$** were run on a Rigaku FRE+ diffractometer equipped with HF Varimax confocal mirrors (100µm focus) and an AFC12 goniometer and HG Saturn 724+ detector. Samples **P1*$_{Zn}$**, **P2$_{Gd2}$** and **P1$_{2H}$** were run on a Rigaku FRE+ diffractometer equipped with VHF Varimax confocal mirrors (70µm focus) and an AFC12 goniometer and HG Saturn 724+ detector. Sample **P1$_{Tb}$** was run on a Rigaku FRE+ diffractometer equipped with VHF Varimax confocal mirrors (70µm focus) and an AFC12 goniometer and HyPix-6000 detector. Sample **P1$_{Y}$** was run on a a Rigaku 007HF diffractometer equipped with Varimax confocal mirrors and an AFC11 goniometer and HyPix-6000 detector. Cell determination and data collection were carried out using CrystalClear[8] (**P1'$_{Dy}$**, **P1*$_{Zn}$**, **P1$_{Zn}$**, **P2$_{Dy2}$**, **P2$_{Gd2}$** and **P1$_{2H}$**) or CrysAlisPro[9] (**P1$_{Dy}$**, **P1$_{Gd}$**, **P1*$_{2H}$**, **P1*$_{Dy}$**, **P1$_{Tb}$**, **P1$_{Y}$** and **P2$_{Tb2}$**). With the data reduction, cell refinement and absorption correction using CrysAlisPro[9]. Using Olex2[10], the structures were solved with either *SUPERFLIP*[11] (**P1$_{2H}$**), ShelXD[12] (**P1$_{Gd}$**) or ShelXT[13] (the rest) structure solution programs and the models were refined with version 2014/7 of ShelXL[14] using Least Squares minimisation. Hydrogen atom positions were calculated geometrically and refined using the riding model. CDC1565176-1565188 contains supplementary X-ray crystallographic data. This data can be obtained free of charge via http://www.ccdc.cam.ac.uk/conts/retrieving.html, or from the Cambridge Crystallographic Data.

2.2 Structures

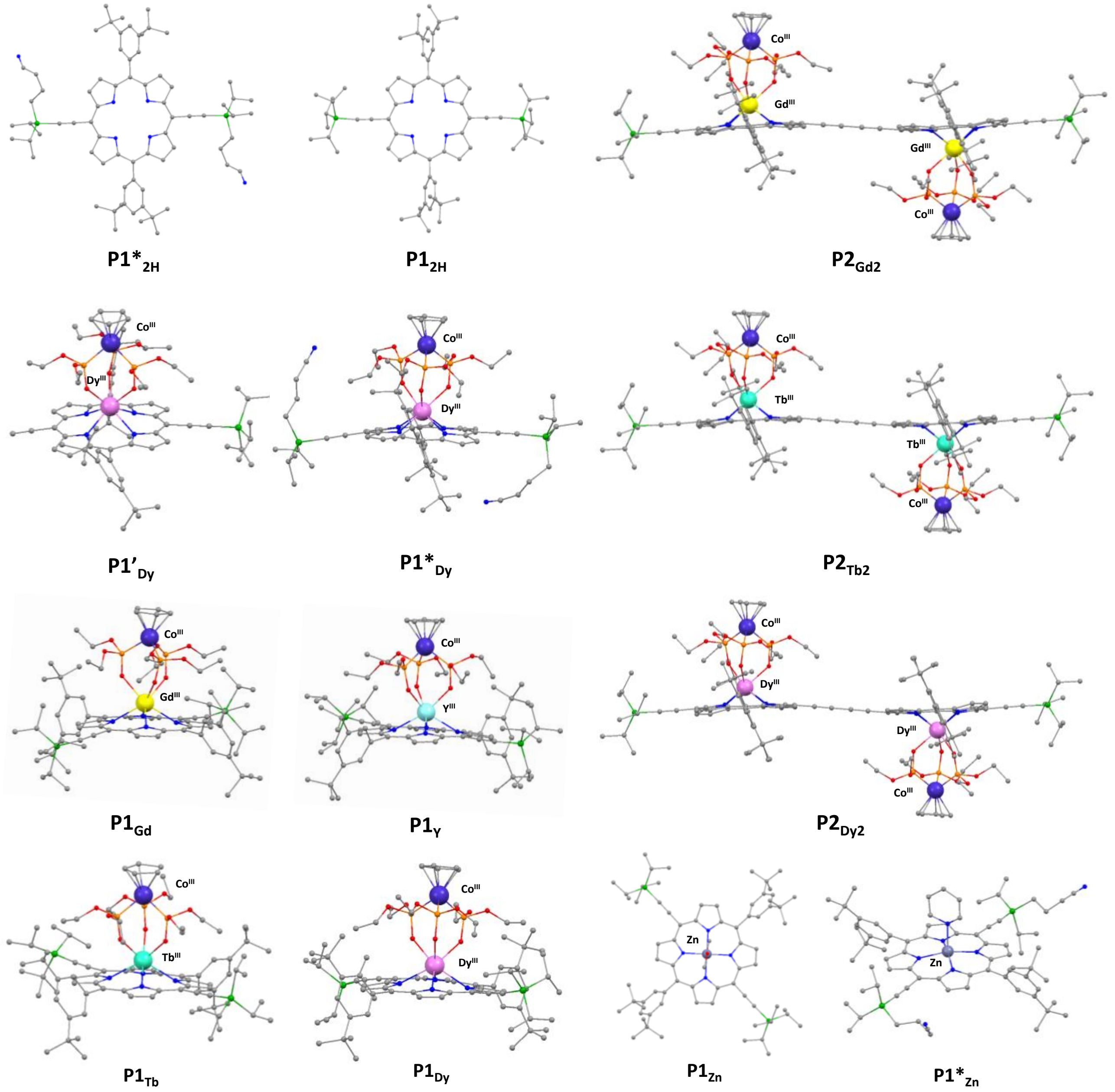

Figure S26. Solid state structures. H atoms and solvent molecules have been removed for clarity. Colour scale: Carbon (grey), oxygen (red), nitrogen (dark blue), phosphorous (orange), cobalt^{III} (purple), dysprosium^{III} (pink), gadolinium^{III} (yellow), terbium^{III} (turquoise), yttrium^{III} (light blue), zink (blue-gray).

2.3 Crystallographic Data

Name	$P1_{2H}$	$P1*_{2H}$	$P1'_{Dy}$	$P1*_{Dy}$	$P1_{Dy}$
CCDC no.	1565183	1565176	1565185	1565181	1565178
Formula	$C_{70}H_{94}N_4Si_2$	$C_{72}H_{92}N_6Si_2$	$C_{78}H_{107}CoDyN_4O_9P_3Si$	$C_{89}H_{125}CoDyN_6O_9P_3Si_2$	$C_{87}H_{127}CoDyN_4O_9P_3Si_2$
$D_{calc.}$/ g cm^{-3}	1.107	1.145	1.333	1.287	1.257
μ/mm^{-1}	0.099	0.102	1.279	1.114	1.116
Formula Weight	1047.67	1097.69	1587.10	1793.46	1743.44
Colour	dark blue	Iridescent dark blue	iridescent blue	dark purple	pale dark blueish green
Shape	blade	plate	cut blade	plate	plate
Size/mm^3	0.130×0.050 ×0.010	0.389×0.209 ×0.017	0.100×0.050×0.03 5	0.180×0.100×0.015	0.296×0.142×0.034
T/K	100(2)	100(2)	100(2)	100(2)	100(2)
Crystal System	triclinic	triclinic	monoclinic	triclinic	triclinic
Space Group	P-1	P-1	$P2_1/c$	P-1	P-1
a/Å	10.7410(11)	10.4966(5)	19.34446(16)	16.1981(3)	16.1487(3)
b/Å	11.0043(11)	12.3975(8)	39.8242(4)	23.3852(4)	19.0993(3)
c/Å	15.1711(14)	13.2265(10)	20.5371(2)	25.4730(5)	30.5159(5)
α/°	69.405(9)	104.289(6)	90	73.821(2)	98.3820(10)
β/°	70.878(9)	99.208(5)	90.4603(9)	89.254(2)	92.0630(10)
γ/°	76.429(9)	101.826(5)	90	86.9650(10)	97.5590(10)
V/Å^3	1571.4(3)	1591.78(18)	15820.8(3)	9253.9(3)	9215.9(3)
Z	1	1	8	4	4
Z'	0.5	0.5	2	2	2
Radiation type	MoK$_\alpha$	MoK$_\alpha$	MoK$_\alpha$	MoK$_\alpha$	MoK$_\alpha$
Θ_{min}/°	2.167	2.306	1.468	2.086	2.365
Θ_{max}/°	25.027	25.026	27.483	27.486	27.560
Measured Refl.	22040	27097	163836	201316	64909
Independent Refl.	5522	5633	36158	42360	64909
Reflections Used	3733	4022	27568	33616	57464
R_{int}	0.0885	0.0828	0.0618	0.0767	.
Parameters	355	399	2201	2472	2124
Restraints	0	8	2414	5794	5367
Largest Peak	0.931	0.757	2.199	4.089	2.187
Deepest Hole	-0.392	-0.489	-0.956	-1.366	-2.050
GooF	1.034	1.024	1.082	1.117	1.090
wR_2 (all data)	0.2183	0.2053	0.1387	0.1815	0.1267
wR_2	0.1940	0.1820	0.1289	0.1694	0.1236
R_1 (all data)	0.1126	0.1048	0.0830	0.1020	0.0626
R_1	0.0738	0.0734	0.0588	0.0782	0.0530

Name	$P1_{Gd}$	$P1_{Y}$	$P1_{Tb}$	$P2_{Gd2}$
CCDC no.	1565179	1565184	1565180	1565188
Formula	$C_{87}H_{127}CoGdN_4O_9P_3Si_2$	$C_{87}H_{127}CoN_4O_9P_3Si_2Y$	$C_{88.5}Cl_3CoH_{130}N_4O_9P_3Si_2Tb$	$C_{156}H_{212}Co_2Gd_2N_8O_{18}P_6Si_2$
$D_{calc.}$/ g cm^{-3}	1.280	1.240	1.314	1.361
μ/mm^{-1}	1.047	3.517	1.134	1.202
Formula Weight	1738.19	1669.85	1867.25	3161.68
Colour	dark green	dark purple	iridescent purple	green
Shape	plate	opaque prism	plate	plate
Size/mm^3	0.180×0.120×0.035	0.190×0.110×0.060	0.319×0.280×0.041	0.190×0.060×0.005
T/K	100(2)	100(2)	100(2)	100(2)
Crystal System	monoclinic	monoclinic	monoclinic	triclinic
Space Group	Cc	$P2_1/c$	$P2_1/c$	P-1
a/Å	16.25392(13)	18.2660(3)	31.5498(4)	10.3106(2)
b/Å	38.80173(18)	14.9061(2)	16.09500(10)	14.6582(3)
c/Å	16.36512(11)	33.9132(5)	19.4714(2)	27.9536(4)
α/°	90	90	90	75.075(2)
β/°	119.1056(10)	104.4550(10)	107.3060(10)	86.248(2)
γ/°	90	90	90	70.983(2)
V/Å^3	9017.84(13)	8941.4(2)	9439.85(17)	3858.65(13)
Z	4	4	4	1
Z'	1	1	1	0.5
Radiation type	MoK$_\alpha$	CuK$_\alpha$	MoK$_\alpha$	MoK$_\alpha$
Θ_{min}/°	2.849	3.183	1.852	2.167
Θ_{max}/°	27.482	70.075	27.485	27.484
Measured Refl.	199380	93429	315944	88673
Independent Refl.	20662	16790	21622	17691
Reflections Used	20621	15304	17246	15807
R_{int}	0.0273	0.0361	0.0656	0.0490
Parameters	1266	1275	1228	1015
Restraints	4110	1416	3508	1386
Largest Peak	0.668	1.360	1.928	3.623
Deepest Hole	-0.451	-0.601	-1.148	-1.068
GooF	1.086	1.061	1.048	1.153
wR_2 (all data)	0.0477	0.1075	0.2681	0.1555
wR_2	0.0477	0.1050	0.2518	0.1512
R_1 (all data)	0.0191	0.0438	0.1022	0.0738
R_1	0.0191	0.0401	0.0872	0.0645
Flack Parameter	0.498(4)			
Hooft Parameter	0.4963(7)			

Name	$P2_{Tb2}$	$P2_{Dy2}$	$P1_{Zn}$	$P1^*_{Zn}$
**CCDC no.**	1565186	1565182	1565187	1565177
**Formula**	$C_{156}H_{212}Co_2N_8O_{18}P_6Si_2Tb_2$	$C_{156}H_{212}Co_2Dy_2N_8O_{18}P_6Si_2$	$C_{74}H_{108}N_4O_4Si_2Zn$	$C_{77.5}H_{96}ClN_7Si_2Zn$
**$D_{calc.}$/ g cm^{-3}**	1.360	1.380	1.148	1.193
**μ/mm^{-1}**	1.257	1.325	0.424	0.462
**Formula Weight**	3165.02	3172.18	1239.19	1282.61
**Colour**	green	green	iridescent purple	iridescent blue-green
**Shape**	plate	plate	block	plate
**Size/mm^3**	0.210×0.080×0.010	0.170×0.110×0.005	0.130×0.110×0.070	0.250×0.065×0.015
***T*/K**	100(2)	100(2)	100(2)	100(2)
**Crystal System**	triclinic	triclinic	monoclinic	triclinic
**Space Group**	P-1	*P*-1	$P2_1/c$	P-1
***a*/Å**	10.30820(10)	10.25340(18)	8.05199(12)	13.1710(4)
***b*/Å**	14.6723(2)	14.6267(3)	13.9466(2)	14.3764(4)
***c*/Å**	27.9524(5)	27.8407(5)	31.9300(5)	19.9456(6)
**α/°**	75.0610(10)	75.0624(16)	90	90.736(2)
**β/°**	86.2080(10)	86.2571(15)	90.6300(13)	91.970(2)
**γ/°**	71.1390(10)	71.1737(16)	90	108.933(3)
**V/Å^3**	3864.76(10)	3817.71(13)	3585.47(9)	3569.14(19)
Z	1	1	2	2
Z'	0.5	0.5	0.5	1
**Radiation type**	MoK$_\alpha$	MoK$_\alpha$	MoK$_\alpha$	MoK$_\alpha$
**Θ_{min}/°**	2.207	2.166	2.846	2.504
**Θ_{max}/°**	27.486	27.484	27.485	27.485
**Measured Refl.**	85977	80838	48760	57370
**Independent Refl.**	17755	17496	8193	16242
**Reflections Used**	16997	15823	7373	11197
**R_{int}**	0.0515	0.0415	0.0296	0.0631
**Parameters**	1197	1052	403	897
**Restraints**	2917	1033	3	986
**Largest Peak**	2.043	1.818	1.338	1.161
**Deepest Hole**	-2.319	-1.070	-0.408	-0.904
**GooF**	1.278	1.102	1.092	1.035
**wR_2 (all data)**	0.1316	0.1126	0.1710	0.2218
**wR_2**	0.1302	0.1096	0.1669	0.1989
**R_1 (all data)**	0.0659	0.0575	0.0629	0.1224
**R_1**	0.0625	0.0499	0.0575	0.814

2.4 Continuous Shape Measure

The Continuous Shape Measures (CShM) software[15] was used to evaluate how close the polyhedral surrounding each lanthanide deviates from the ideal shape. The coordination polyhedron with the lowest CShM value defines the best polyhedron. Values larger than 0.1 are considered chemically significant distortions while values larger than about 3 indicate important distortions.

Below is the CShM for the coordination polyhedra of each lanthanide in the structure of complex **$P1_{Dy}$, $P2_{Dy2}$, $P1_{Tb}$, $P2_{Tb2}$, $P1_{Gd}$, $P2_{Gd2}$** comparted to known 7-vertex polyhedral.

	Heptagon (D7h)	Hexagonal pyramid (C6v)	Pentagonal bipyramid (D5h)	Capped octahedron (C3v)	Capped trigonal prism (C2v)	Johnson pentagonal bipyramid J13 (D5h)	Johnson elongated triangular pyramid J7 (C3v)
**$P1_{Dy(a)\_1}$**	35.378	19.198	6.035	0.814	0.82	9.728	20.895
**$P1_{Dy(a)\_2}$**	36.331	18.432	7.138	1.634	0.956	10.975	20.998
**$P1_{Dy(b)}$**	35.853	19.252	6.38	0.281	1.717	10.009	20.636
**$P2_{Dy2\_1}$**	35.915	19.275	5.861	0.524	1.635	9.706	20.612
**$P2_{Dy2\_2}$**	34.903	19.598	6.208	0.766	1.076	9.712	19.46
**$P1_{Tb\_1}$**	36.323	18.778	5.842	0.681	1.611	9.838	20.555
**$P1_{Tb\_2}$**	36.368	18.741	6.489	1.278	1.126	10.276	21.01
**$P2_{Tb2\_1}$**	35.824	19.128	5.916	0.554	1.696	9.777	20.727
**$P2_{Tb2\_2}$**	34.871	19.453	6.217	0.73	1.235	9.744	19.547
**$P1_{Gd\_1}$**	34.669	19.377	6.97	1.262	0.637	10.675	20.939
**$P1_{Gd\_2}$**	35.068	19.305	6.676	1.8	0.895	10.681	20.251
**$P1_{Gd\_3}$**	35.418	18.713	6.241	1.117	0.95	10.368	20.749
**$P2_{Gd2\_1}$**	35.664	19.153	5.919	0.563	1.736	9.743	20.628
**$P2_{Gd2\_2}$**	34.764	19.357	6.257	0.743	1.268	9.823	19.64

* **$P1_{Dy(a)}$/ $P1_{Dy(b)}$** represent the two different molecules present in the asymmetric unit for **$P1_{Dy}$.** The _1, _2 and _3 represent the different parts of disorder of the phosphine ligands in the molecules.

3 Squid Measurements

3.1 Experimental Details

For all samples, SQUID analyses were performed on crushed polycrystalline samples wrapped in a polyethylene membrane. The direct current (dc) magnetic susceptibility measurements were obtained using a Quantum Design SQUID magnetometer MPMS-XL7. Dc measurements were taken in the temperature range of 1.8 and 300 K for fields ranging from -7 to 7 T. For the Dy and Tb-containing samples alternating current (ac) magnetic susceptibility measurements were carried out under an oscillating ac field of 4 Oe and ac frequencies ranging from 1 to 1500 Hz. Diamagnetic corrections were applied for the sample holder and for the core diamagnetism from the sample, calculated from Pascal constants.

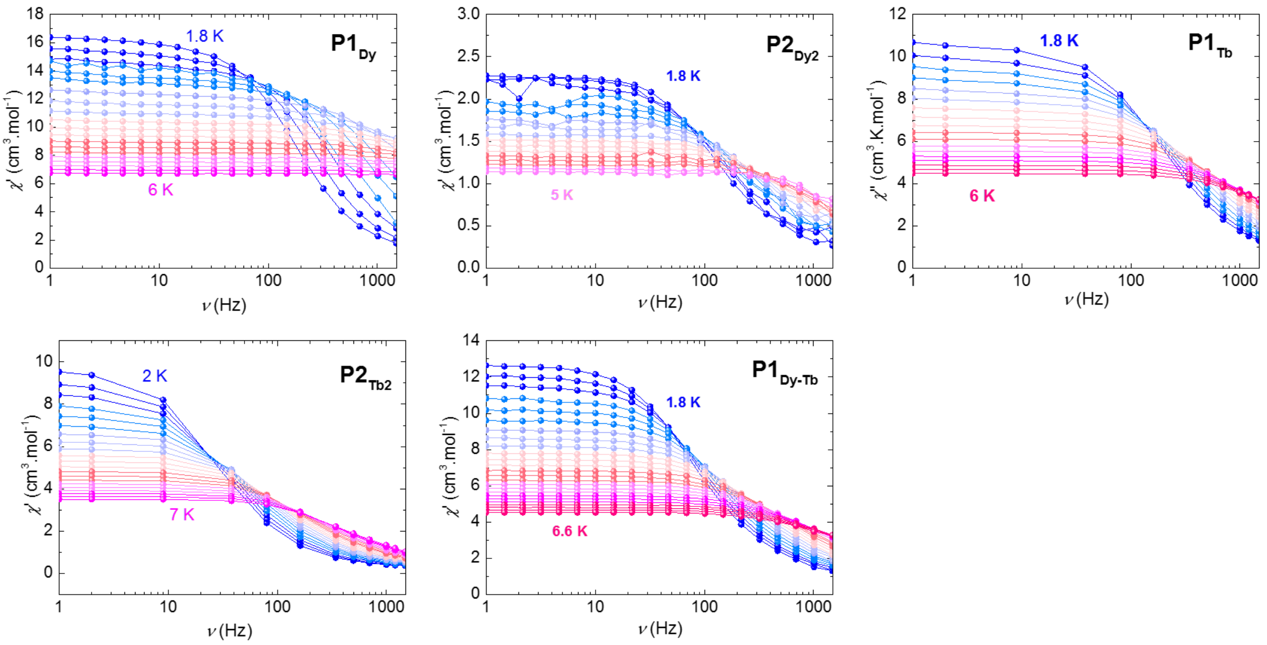

Figure S27. In-phase (χ') magnetic susceptibility of **P1$_{Dy}$**, **P2$_{Dy2}$**, **P1$_{Tb}$**, **P2$_{Tb2}$**, and **P1$_{Dy\text{-}Tb}$**. All measurements were taken crushed polycrystalline samples under an applied dc field of 2000 Oe and an ac field of 4 Oe.

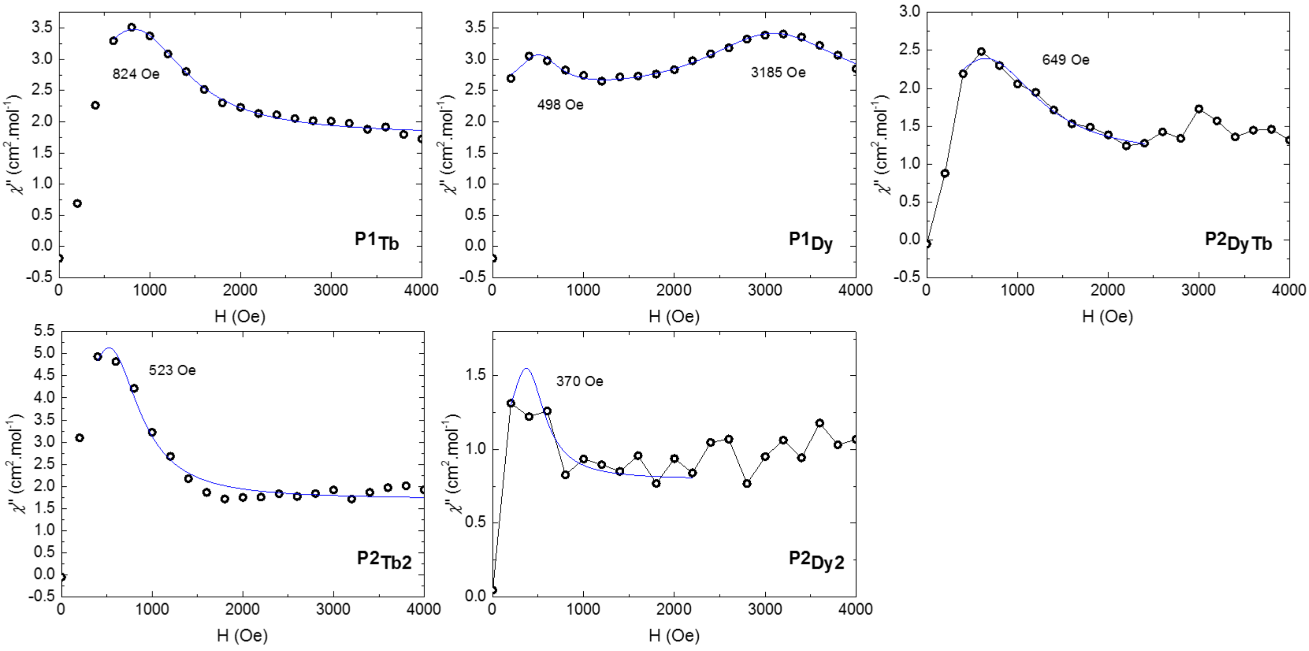

Figure S28. Variable field χ'' magnetic susceptibility of **P1$_{Tb}$**, **P2$_{Tb2}$**, **P1$_{Dy}$**, **P2$_{Dy2}$** and **P2$_{Dy\text{-}Tb}$** at 2 K and 1500 Hz. The blue line is a Lorenz Fit to the data. As expected we see 2 peaks in the field dependence of **P1$_{Dy}$**, one for each of the two molecules in the asymmetric.

4 Electron Paramagnetic Resonance Spectroscopy

4.1 Methods

EPR data were collected in the Centre for Advanced ESR (CAESR) in the Department of Chemistry, University of Oxford. All X-band data were obtained on a Bruker EleXsys E680. Q-band data was acquired using a Bruker Elexsys E580. An Oxford Instruments CF9350 cryostat was used to maintain the sample temperature and an Oxford Instruments ITC503 to control it. As microwave resonators, we used a Bruker ER4118X-MS3-W1 on the EleXsys E680 and Bruker EN510702 on the EleXsys E580. Magnetic fields were generated with an electromagnet. For measurements at X-band frequency, the sample was dissolved with a concentration of 1 mmol/l in Toluene/Dichloromethane (1:1). For Q-band frequency, we prepared a 1 mmol/l solution in Acetonitrile/2-Methyltetrahydrofuran.

FID-detected absorption spectra

Free induction decay (FID)-detected absorption spectra were obtained by excitation of the system with a long π-pulse (800 ns). Therefore, a FID signal can be recorded after the receiver protection switch. Measurements were performed at cryogenic temperatures which enables a strong signal.

Phase-memory and quantum coherence times

The phase-memory time was obtained using a Hahn-echo sequence, π/2 - τ - π - τ - echo. A π/2-pulse turns the spin orientation into the xy-plane, where they start to interact freely. During this evolution period, the spins interact with their environment (such as nuclear spin bath or spin-spin interactions) and begin to dephase. After a time-delay τ, we apply a π-pulse and thus after another time τ, spins recombine and yield a signal intensity (echo). Since decoherence occurs over both periods of τ, the data is fit with a dependence of the signal intensity versus time 2τ using

$$Y(\tau) = Y_0 e^{-\left(\frac{2\tau}{T_m}\right)^x},$$

where Y corresponds to the signal intensity, T_m to the phase-memory time and x is a stretching factor to account for the composition of the factors that influence dephasing. At Q-band frequency, we used pulse durations of 16 ns and 32 ns for the π/2 and π pulses, respectively. The magnetic field at Q-band frequency was set to 1281.3 mT. At X-band frequency, we used longer pulse durations of 500 ns and 1000 ns for the π/2 and π pulses, respectively, to remove modulation effects in the spectrum.

Spin-lattice relaxation times

The spin-lattice relaxation time T_1 was obtained using the inversion recovery technique, π - t - π/2 - τ - π - τ - echo. A π-pulse flips the orientation of the spins, and after a time t the recovered spin intensity is measured with a Hahn-echo sequence as shown above. Data was fitted using a sum of two exponential functions to account for spectral diffusion. The part with the larger value of T_1 was identified to be related with spin-lattice relaxation. We used pulse durations of 16 ns and 32

ns for the π/2 and π pulses, respectively, and a fixed dephasing time of τ = 300 ns. Magnetic field at Q-band frequency was set to 1281.3 mT.

4.2 Q-band spectrum

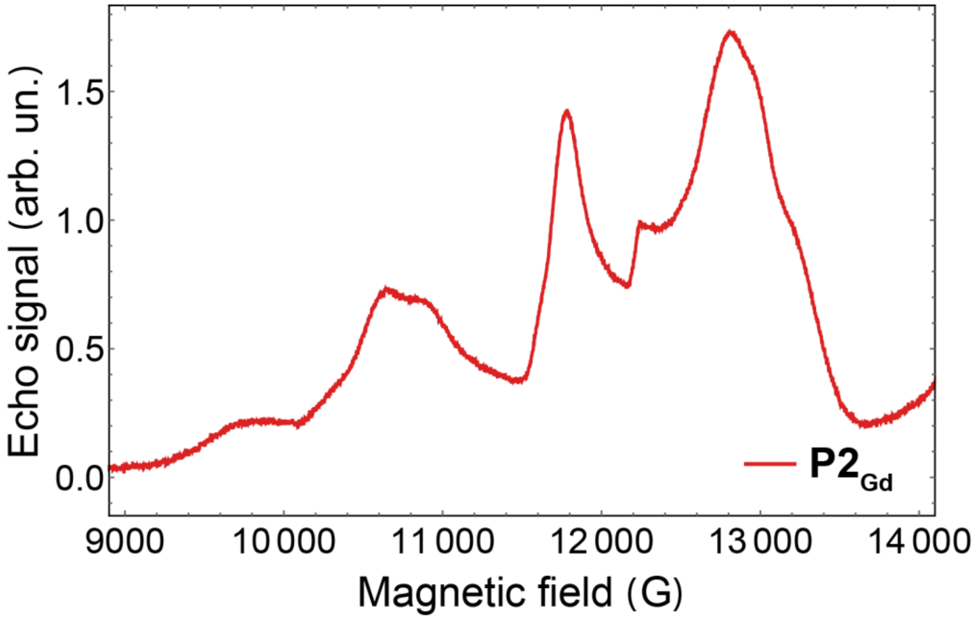

Figure S29. Central EPR transitions within the FID-detected absorption spectrum of **P2Gd₂** at Q-band frequency (33.9926 GHz) and 10 K, from integrating the FID of a 800 ns pulse.

4.3 Spin-lattice relaxation at Q-band frequency

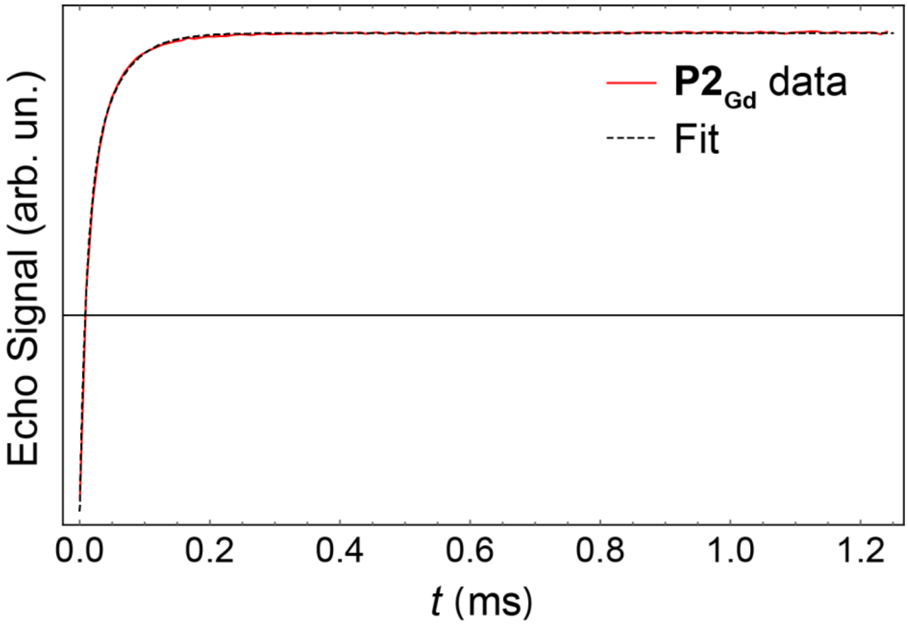

Figure S30. Inversion recovery behavior of $\mathbf{P2_{Gd2}}$ at Q-band frequency (≈34 GHz), 10 K and 1281.3 mT. The fitted relaxation times are 10.0 μs and 43.4 μs, accounted for spectral diffusion and spin-lattice relaxation time T_1, respectively.

4.4 Phase-Memory Time T_m at Q-band frequency

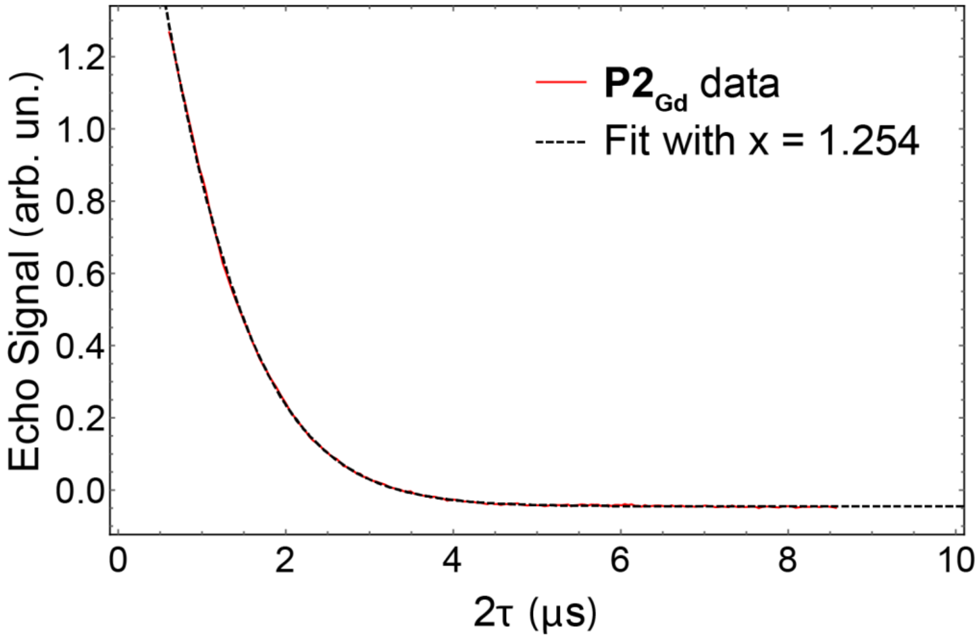

Figure S31. Echo signal after the Hahn-echo sequence of **P2$_{Gd2}$** at Q-band frequency (≈34 GHz), 10 K and 1281.3 mT. We found x = 1.254 and Tm = 2297 ns. A slightly worse, but better comparable fit is obtained when the stretch factor is fixed to 1, giving T_m = 1786 ns.

4.5 Quantum coherence times versus field at X-band frequency

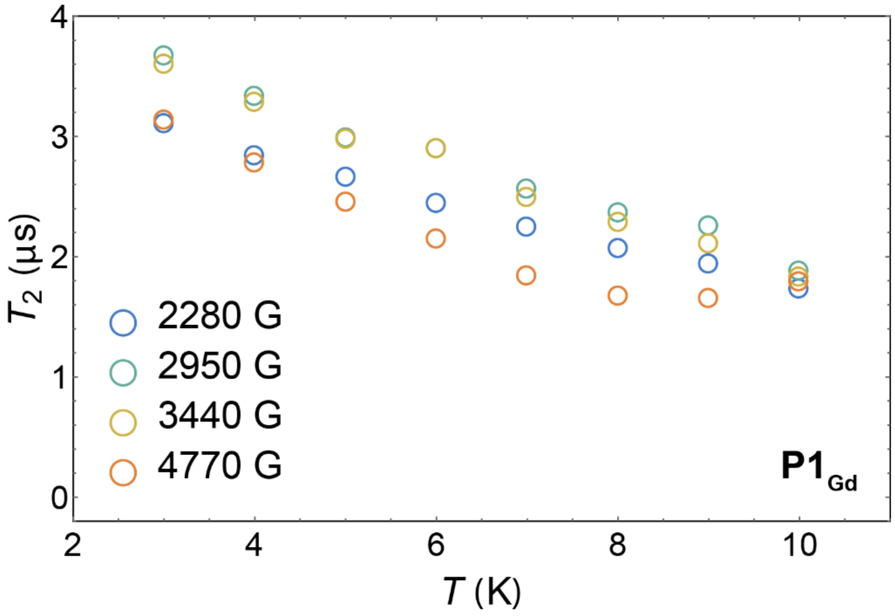

Figure S32. Obtained quantum coherence times T_2 for **P1$_{Gd}$** at different magnetic fields and different temperatures at X-band frequency (≈9.5 GHz).

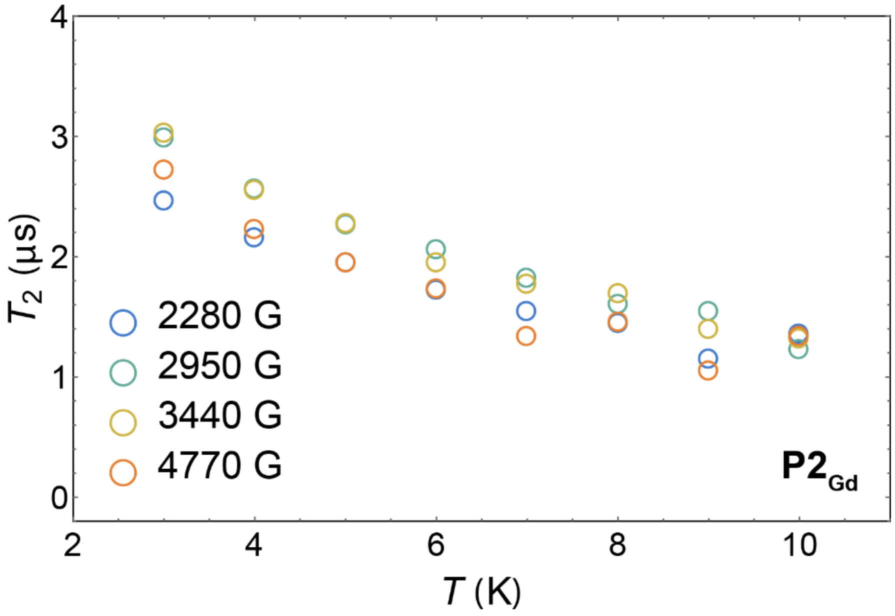

Figure S33. Obtained quantum coherence times T_2 for $\mathbf{P2_{Gd2}}$ at different magnetic fields and different temperatures at X-band frequency (≈9.5 GHz).

5 References

(1) M. J. Plater, S. Aiken and G. Bourhill, G. *Tetrahedron,* 2002, **58**, 2405.

(2) M. J. Plater, S. Aiken and G. Bourhill, *Tetrahedron,* 2002, **58**, 2415.

(3) V. Harder, E. Dubler and H. Werner, *J. Organomet. Chem.,* 1974, **71**, 427.

(4) F. Gao, M.-X. Yao, Y.-Y. Li, Y.-Z. Li, Y. Song and J.-L. Zuo, *Inorg. Chem.,* 2013, **52**, 6407.

(5) W. Kläui, H. Neukomm, H. Werner, G. Huttner, *Chem. Ber.,* 1977, **110**, 2283.

(6) W. Kläui, *Angew. Chem. Int. Ed.,* 1990, **29**, 627.

(7) S. J. Coles and P. A. Gale, *Chem. Sci*., 2012, 3, **3**, 683.

(8) Rigaku, *CrystalClear- SM Expert 3.1 b27,* **2013.**

(9) CrysAlisPro Software System, Rigaku Oxford Diffraction, Yarnton, Oxford, UK, **2015**.

(10) O. V. Dolomanov, L. J. Bourhis, R. J. Gildea, J. A. K. Howard, H. Puschmann, *J. Appl. Cryst.*, 2009, **42**, 339.

(11) L. Palatinus and G. Chapuis, *J. Appl. Cryst*., 2007, **40**, 786.

(12) G. M. Shedrick, *Acta. Cryst*, 2008, **A64**, 339.

(13) G. M. Sheldrick, *Acta. Cryst.*, 2015, **A71**, 3.

(14) G. M. Sheldrick, *Acta. Cryst.*, 2015, **C27**, 3.

(15) M. Llunell, D. Casanova, J. Girera, P. Alemany, S. Alvarez, SHAPE, Continuous Shape Measures Calculation, version 2.0; Universitat de Barcelona: Barcelona, Spain, 2010.
